# FFL-IDS: A Fog-Enabled Federated Learning-Based Intrusion Detection System to Counter Jamming and Spoofing Attacks for the Industrial Internet of Things

**DOI:** 10.3390/s25010010

**Published:** 2024-12-24

**Authors:** Tayyab Rehman, Noshina Tariq, Farrukh Aslam Khan, Shafqat Ur Rehman

**Affiliations:** 1 Department of Information Engineering, Computer Science, and Mathematics, University of L’Aquila, 67100 L’Aquila, Italy; tayyab.rehman@graduate.univaq.it; 2 Department of Artificial Intelligence and Data Science, National University of Computer and Emerging Sciences, Islamabad 44000, Pakistan; noshina.tariq@isb.nu.edu.pk; 3 Center of Excellence in Information Assurance, King Saud University, Riyadh 11653, Saudi Arabia; 4 Stony Brook Institute at Anhui University, Hefei 230000, China; 24401@ahu.edu.cn

**Keywords:** convolutional neural network, federated learning, industrial internet of things, jamming attack, spoofing attack, network intrusion detection

## Abstract

The Internet of Things (IoT) contains many devices that can compute and communicate, creating large networks. Industrial Internet of Things (IIoT) represents a developed application of IoT, connecting with embedded technologies in production in industrial operational settings to offer sophisticated automation and real-time decisions. Still, IIoT compels significant cybersecurity threats beyond jamming and spoofing, which could ruin the critical infrastructure. Developing a robust Intrusion Detection System (IDS) addresses the challenges and vulnerabilities present in these systems. Traditional IDS methods have achieved high detection accuracy but need improved scalability and privacy issues from large datasets. This paper proposes a Fog-enabled Federated Learning-based Intrusion Detection System (FFL-IDS) utilizing Convolutional Neural Network (CNN) that mitigates these limitations. This framework allows multiple parties in IIoT networks to train deep learning models with data privacy preserved and low-latency detection ensured using fog computing. The proposed FFL-IDS is validated on two datasets, namely the Edge-IIoTset, explicitly tailored to environments with IIoT, and CIC-IDS2017, comprising various network scenarios. On the Edge-IIoTset dataset, it achieved 93.4% accuracy, 91.6% recall, 88% precision, 87% F1 score, and 87% specificity for jamming and spoofing attacks. The system showed better robustness on the CIC-IDS2017 dataset, achieving 95.8% accuracy, 94.9% precision, 94% recall, 93% F1 score, and 93% specificity. These results establish the proposed framework as a scalable, privacy-preserving, high-performance solution for securing IIoT networks against sophisticated cyber threats across diverse environments.

## 1. Introduction

Internet of Things (IoT) is the networking infrastructure of connected computing devices, applications, and other systems that can automatically perform critical functions with little to no human intervention [1]. Industries’ adoption at an unprecedented pace has caused severe security issues. Thus, practical approaches are necessary to ensure data privacy, confidentiality, and availability. IoT and Industrial Internet of Things (IIoT) are related concepts but differ markedly in their requirements, work actions, legal considerations, and applications [2]. However, IIoT has been confined to industrial manufacturing with higher compliances compared to consumer IoT, with the increased complexity of the system. The broad proliferation of IoT technologies within the last decade raises, at the very foundation, the concept of IoT security, which focuses on managing diverse and heterogeneous protocols and data integrity across distributed systems [3]. Generally, IoT devices have inherent limitations related to energy constraints, limited memory, and low computational power, making it even harder to secure these systems. These pose a challenge in industrial environments because IIoT applications are fundamentally different, with critical functionalities, and even minor operational disruptions may have significant financial and safety implications [4].

The IIoT is an evolution of the IoT that uses integrated cloud and edge computing to automate and optimize industrial operations [5]. IIoT relies on networked manufacturing devices to gather, process, and analyze data, significantly enhancing production efficiency. It points out that a multi-layered architecture is essential for IIoT, involving distinct layers such as sensing, edge processing, networking, and cloud-based analytics. It necessitates layers that require frictionless communication protocols, a fault-tolerant system, and mechanisms for end-to-end security. IIoT has revolutionized the old way of manufacturing. It helps execute intelligent, data-driven operations since it is enabled through interconnected sensors and networked systems. Predictive maintenance, real-time decision making, and resource optimization can be managed through it [6,7]. However, specific challenges need to be addressed, for instance, interoperability between legacy systems and modern IoT devices; a significant challenge is the financial burden of implementing and managing large-scale IIoT solutions, as stated in [8]. The adoption rate of IIoT is expected to grow at a compound annual growth rate of over 20%. The applications in manufacturing, energy, and healthcare are a few examples. Such data have been included in strengthening the argument for the industrial importance of IIoT [9]. An industry can have long-term growth and competitive advantage in the digital economy if suitable real-time data and automation insights maximize resource utilization while minimizing risks [10]. Such improved connectivity also brings along considerable risks. Network Intrusion Detection System (NIDS) is essential for countering such risks when it monitors and analyzes network traffic for malicious activity [11].

NIDS relies on complex algorithms to discover and prevent varied cyber attacks. DL techniques have become a potent approach to intrusion detection enhancement within the last few years. DL algorithms perform very well in processing high-dimensional data and discovering subtle patterns that indicate malevolent behavior [12]. The centralized nature of DL-based Intrusion Detection System (IDS) introduces privacy concerns, especially when handling sensitive network traffic data. Some privacy-preserving solutions, like federated learning, have also been proposed to handle these issues, which enables collaborative model training without exposing raw data [13]. Nonetheless, the effectiveness of DL-based NIDS depends on the high quality and large scale of datasets for training purposes [14]. Centralized data collection suffers from scalability issues due to exponential growth in network traffic, as well as issues of privacy. Emerging research highlights the requirement for hybrid solutions that combine centralized and edge-based processing to balance privacy, scalability, and performance in IDS [15]. Given these challenges, this study discusses novel approaches to capitalize on the capabilities of IIoT and mitigate its security vulnerabilities, focusing on integrating DL methods with privacy-preserving approaches.

Federated Learning (FL), a framework initially proposed by Google, is a paradigm shift in Machine Learning (ML) that places significant emphasis on protecting data privacy and security [16]. This paradigm has been applied in various sectors, such as mobile device prediction, medical health, and recommendation systems. The core idea is the development of ML models using data dispersed over multiple devices, focusing on minimizing the potential dangers associated with exposure to the data. Historically, the practice of ML involved gathering data in a centralized manner for training models [17]. Nevertheless, there are notable differences in FL’s architecture. The system consists of a server and numerous clients, where the server refrains from directly collecting raw data but can gather model parameters. The server assumes the role of a conductor, coordinating client activity in training, whereby each client retains its localized training dataset. The training of individual models on the client side utilizes local data, and subsequently, the generated model parameters are transmitted to the server, either in an encrypted or unencrypted manner [18]. The server then collects these parameters using average or weighted averaging methods and delivers them back to each client for subsequent training iterations [19]. This novel methodology facilitates cooperative learning from dispersed origins and guarantees data confidentiality and protection, rendering it a highly promising resolution for detecting network breaches in the IIoT framework.

FL has emerged as a feasible alternative in response to data privacy concerns. The state of Florida enables the collaborative training of a shared model through the utilization of FL, a framework that facilitates the transmission of solely model parameters to cloud servers [20]. This approach mitigates the risk of exposing raw data during training. Nevertheless, if the quantity of users escalates, this methodology may provide a constraint, resulting in potential deterioration of network performance and increased use of resources. This study addresses the issues above by proposing an innovative NIDS that utilizes Fuzzy Logic in conjunction with a Convolutional Neural Network (CNN). The proposed system also features a layered architecture using fog computing [21]. In IIoT, the above system can enhance intrusion detection performance while ensuring privacy preservation, real-time responsiveness, and scalability. The proposed approach forms the ground for building solid and practical IDS for edge computing and the IIoT. This approach will consider some essential aspects, including data privacy, latency, and resource constraints.

### Key Contributions

This paper provides several critical improvements in intrusion detection in the context of the IIoT.

- Proposed FFL-IDS Framework: A Fog-enabled Federated Learning-based Intrusion Detection System (FFL-IDS) that develops a CNN-based system to detect jamming and spoofing attacks in IIoT networks while preserving data privacy.
- Incorporation of Fog Computing: It employs fog computing to preprocess IIoT data in a real-time fashion, reducing latency by enabling low-latency intrusion detection closer to the point of data generation.
- Privacy-Preserving Collaboration: The framework allows multiple IIoT stakeholders to collaboratively train deep learning models without sharing raw data, ensuring privacy preservation across distributed environments.
- Comprehensive Validation: Experimental evaluations are conducted on two datasets, Edge-IIoTset, designed for IIoT-specific scenarios, and CIC-IDS2017, representing diverse network environments. The results demonstrate superior detection accuracy, precision, recall, F1 score, and specificity performance.
- Scalability and Robustness: The proposed approach obtains better performance than existing works by achieving 93.4% accuracy on Edge-IIoTset and 95.8% accuracy on CIC-IDS2017, demonstrating its adaptability and robustness across different datasets.

The rest of the paper is organized as follows: Section 2 presents the background. Section 3 discusses the related work. Case studies are examined in Section 4. The proposed architecture is specified in Section 5 and our methodology is outlined in Section 6. Section 7 details the dataset preparation and analysis. Implementation details are provided in Section 8. Evaluation and results are presented in Section 9. Section 10 details the challenges in this study and a discussion. Finally, Section 11 concludes the paper with potential future research directions.

## 2. Background

This section explains the IIoT and its potential to transform various industries while outlining its security risk. It breaks down the IIoT applications of manufacturing, healthcare, and logistics and emphasizes the need for high-quality security solutions to fight against security threats. This lays the foundation for the present research by highlighting the importance of securing IIoT systems for operational and scalability purposes.

### 2.1. Industrial Internet of Things

**Figure 1:**
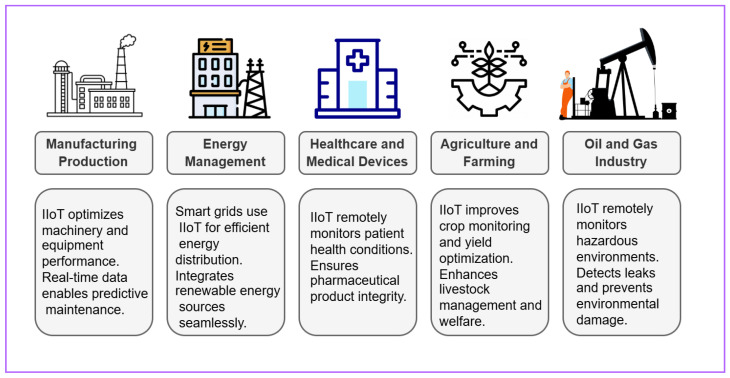
IIoT applications.

The IIoT is a revolutionary technological transformation in manufacturing, logistics, and other commercial and industrial settings. It performs its tasks by gathering data in real time from a vast array of sensors and devices, enabling massive data analysis and decision making [22]. Unlike general IoT applications, IIoT focuses on improving operational efficiency, addressing energy demands, and optimizing logistics in industrial environments. It is reshaping business processes across several sectors. It improves manufacturing operations and quality assurance [23]. It paves the way for data-driven care and remote patient monitoring in the medical field. It improves supply chain management efficiency in the logistics industry [24]. The possibilities of IIoT are enormous, but so are the corresponding security risks. IIoT also introduces significant security risks, requiring robust measures such as intrusion detection, secure communication, and data privacy. Figure 1 demonstrates various IIoT applications, such as oil and gas operations, healthcare production, and energy management. These applications emphasize industrial-scale requirements, such as fault tolerance, real-time decision making, and system scalability. The research community aspires to construct reliable and durable IIoT ecosystems by creating cutting-edge solutions like intrusion detection, data privacy, secure communication, and interoperability.

The IIoT combines obsolete industrial gear with new Internet-connected devices, creating an ecosystem susceptible to security risks [25]. Many industrial systems were not developed with cybersecurity, making them difficult to safeguard and frequently leaving known vulnerabilities unresolved due to industrial equipment’s extended operational lifecycles [26]. IIoT networks face a wide range of cyber threats, including Denial of Service (DoS) and Man-in-the-Middle (MitM) attacks, as well as ransomware and data breaches. Intrusion assaults, in which unauthorized actors obtain network access, are severe owing to the potential for physical harm [3]. These might be passive, including monitoring and gathering data without interfering with system operations, or active, involving the manipulation or interruption of operational processes [27]. Such incursions have far-reaching consequences, ranging from economic losses and data breaches to potentially catastrophic situations affecting physical safety and the environment. As a result, defending IIoT systems from these attacks is vital for data protection, critical infrastructure, and human safety [28]. Table 1 provides a thorough overview of the cyber threats that IIoT systems face, categorizing and describing these assaults and their potential network effect.

### 2.2. Network Intrusion Detection

**Figure 2:**
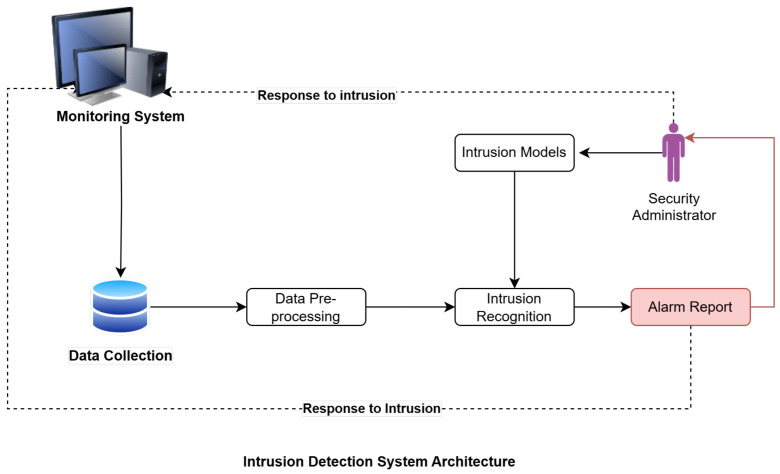
Intrusion Detection System (IDS) architecture for threat detection and response.

As IIoT networks grow in scale and complexity, ensuring robust data privacy, real-time responsiveness, and low latency becomes essential. Integrating NIDS with edge computing and FL enhances security by decentralizing intrusion detection while preserving data privacy [33]. The importance of NIDS grows in the complex IIoT ecosystem, where uninterrupted communication and data interchange are crucial to vital infrastructure and industrial operations [34]. However, as IIoT networks expand in size, complexity, and connection, they raise new and varied security problems. Due to the sensitive nature of industrial data, several essential requirements must be met for IIoT ecosystems to thrive [35]. These include data privacy, real-time responsiveness, and scalability. Edge computing integration guarantees low latency real-time response capabilities, and FL allows multiple entities to train intrusion detection models collaboratively without compromising data privacy [36]. The importance of privacy-preserving methods in strengthening the security infrastructure of IIoT environments has also grown [33]. Together, these initiatives strengthen the data security of IIoT and provide businesses with more agency as they adapt to new technologies. Figure 2 shows an IDS structured process, showing essential steps such as data collection, preprocessing, intrusion recognition, and response mechanisms. It stresses the importance of intrusion models and warning reporting in letting security staff know about possible threats so that they can take action quickly to lower risks.

### 2.3. Federated Learning

**Figure 3:**
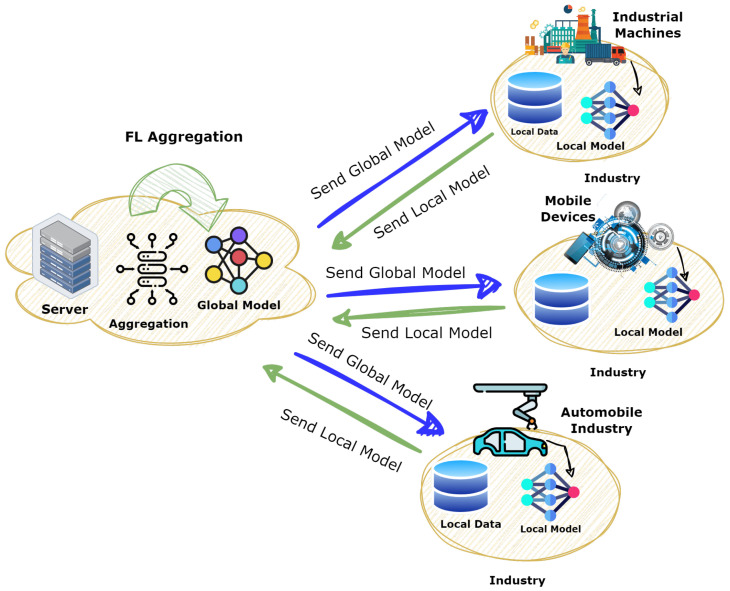
Representation of an FL model.

The problems encountered in this IIoT scenario are well-suited to FL. In this case, FL enables the training of ML models on these devices locally, rather than sending the data from multiple IoT devices centrally so that the privacy related to data is maintained [19]. Instead of sending the original data, the models’ updates or gradients are sent to the server during training. This server summarizes all these changes into a single global update. This method gives various IIoT advantages. It reduces private or massive data requirements to be sent through the networks by saving time and space [37]. It supports scalable and secure anomaly detection with good decentralization and is necessary for various industrial systems and dispersed data sources. It also supports real-time processing at the edge for immediate insights and choices. FL promises to provide better safety, anomaly detection, and predictive maintenance in operation without compromising privacy and real-time responsiveness because of its scalability and adaptability to the dynamic nature of the environments of IIoT [38]. Figure 3 represents the FL Model representation.

### 2.4. Convolutional Neural Networks

**Figure 4:**
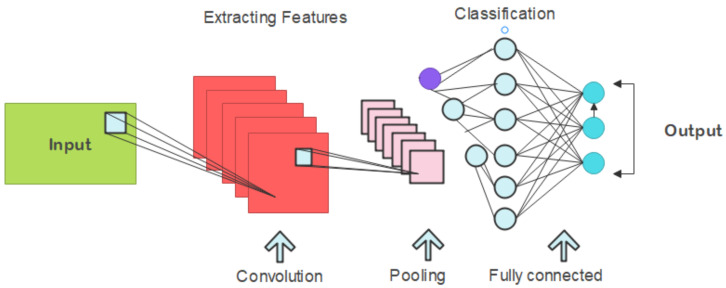
Representation of a CNN model.

CNNs are primarily used in network intrusion detection domains. Being DL models, they are also remarkably efficient in image processing and pattern recognition [39]. However, several key elements must be used in conjunction with CNNs. The convolutional layers utilize filters for rapidly scanning input data for patterns, reducing data dimensionality by the pooling layers while preserving pertinent characteristics intact. Fully connected layers are used in predicting and classifying [40]. They are trained in network intrusion detection using the tagged benign and malicious traffic data. During their operation, they analyze the real-time network traffic, looking for signs of possible attacks. IDSs that utilize CNNs may adapt and learn to newer threats through retraining with new data [41]. These devices reduce unnecessary warnings when they detect accurate threats using a calibration that minimizes false positives and negatives. Essentially, CNNs are feasible network security tools. The pattern recognition abilities of the CNNs can be leveraged to improve the real-time identification and mitigation of security risk factors in network traffic. The findings using this method thus enable industrial systems that have various and widely spread data sources to scale anomaly detection efficiently and securely. Figure 4 depicts the structural arrangement of the CNN model we employ in this work. This illustration explains how the CNN layers are arranged and connected differently. Our model has been designed so that depth and efficiency in the CNN architecture can effectively capture even complex patterns in the input data.

## 3. Related Work

This section critically analyzes the literature on intrusion detection algorithms operating in traditional and IIoT networks with a special emphasis on ML- and DNN-based approaches. While conducting the review, the following trustworthy scientific databases were consulted: IEEE Xplore, Scopus, Web of Science, ScienceDirect, and ACM Digital Library. The literature search was conducted against keywords such as ‘intrusion detection’, ‘machine learning’, ‘deep learning’, ‘IIoT security’, and ‘cybersecurity algorithms’. Papers were chosen considering their publication in high-impact journals and conferences, relevance to intrusion detection in IIoT, and publication dates between 2020 and 2024, ensuring that the focus is on recently developed advancements. Examples of reviewed articles are studies on intrusion detection using deep learning in IIoT environments [35,42], hybrid models for intrusion detection [12,13], and trust-based mechanisms to enhance IIoT security [41,43]. These sources provide a solid foundation for understanding the advancements and challenges in this domain.

Xu et al. [44] examined four separate assault models and extensively evaluated their effects on wireless communication between a sender and receiver. This study successfully tackles the issue of verifying the existence of jamming signals, acknowledging the limitation that a solitary measurement may not yield a reliable confirmation. In order to address this matter, the study proposes two improved methodologies that incorporate consistency checks. The initial system uses signal strength as a reactive measure of consistency, whereas the second scheme capitalizes on location information. The detection of threats in 802.11 networks has been addressed by Oscar et al. [45] with the introduction of a random forest-based technique. The results of their study suggest that this approach has superior performance compared to other established methodologies.

Bansal et al. [46] introduced an intrusion detection system (IDS) based on anomaly detection with the utilization of three deep learning (DL) classifiers, namely, Clustering, LSTM-stimulated Neural Networks, and Recurrent Neural Networks. It was designed explicitly for IIoT applications and was tested using the ISCX and CTU-13 datasets. The results obtained had an accuracy of 98.8%, 98.39%, and 83.09% for Clustering, NN-LSTM, and RNN, respectively. The ISCX is used widely in industrial control systems’ cybersecurity, while the CTU-13 dataset primarily focuses on botnet traffic in machine learning model training. Sharma et al. [47] proposed a machine learning-based mechanism that finds novel malware using network flow-based analysis. Their database consists of 11,688 malware and 4006 benign samples from the Malicia project, and other noise-generating systems connected with the same network. Different ML classification techniques were applied to evaluate the network flow: RF, LMT, FT, and J48 classifiers.

The study by Javeed et al. [48] specified an intelligent framework for IIoT threat mitigation. The system employed DL in conjunction with SDN techniques. The authors adopted a hybrid model, namely the Cu-LSTMGRU + Cu-BLSTM model, to enhance the effectiveness of threat detection. This hybrid model was compared to Cu-DNNLSTM and Cu-DNNGRU models. All models were trained and experimented using the N-BaIoT dataset [49]. The Cu-LSTMGRU + Cu-BLSTM model performed best and achieved an accuracy of 99.45%, precision of 99.34%, recall of 98.49%, and F1 score of 99.47%.

Baig et al. [50] provided an innovative approach for identifying DoS attacks in smart IoT sensors. The authors had set up a dedicated IoT testbed using the MQTT protocol, which included various DoS attack techniques abnormal to this protocol. To build their dataset, the authors took several packet-level attributes, including source and destination IP addresses, port numbers, framing length, IP packet length, TCP segment length, and header length [51]. The approach demonstrated that the classifier using A1DE/A2DE attained a better classification accuracy as it experimented upon both the custom-built dataset and the BoT-IoT testbed.

Jing and Chen [52] evaluated an SVM-based, IIoT-specific NIDS using the UNSW-NB15 dataset. The NIDS was explicitly designed to cater to the distinct attributes of IoT networks. Its primary emphasis was on essential performance indicators such as accuracy, detection rate, and false positive rate. The conducted trials encompassed both binary and multiclass classification tasks. The SVM-based NIDS has a noteworthy accuracy rate of 85.99% for binary classification. The study in [53] presented a sophisticated IDS that used a multi-objective feature selection technique. This technique employed a specific variation of the GA in conjunction with the LR algorithm. The efficacy of this methodology was evaluated by utilizing diverse ML techniques, such as RF, and the assessment of model performance was conducted utilizing datasets such as UNSW-NB15.

Existing methods prioritize binary classification or rely on datasets that do not include IoT traces. The lack of dependable IoT datasets incorporating attack traffic is partly to blame for this deficiency. We have created a novel intrusion detection method using DL and generic features at the packet level. We describe an FL model based on a Fog-enabled CNN for attack detection in IIoT environments, which can be used for binary and multiclass classification. Table 2 provides a deep analysis comparing different intrusion detection techniques designed for IIoT networks.

## 4. Case Studies

This section discusses two major case studies concerning the significant security challenges in IIoT environments. Case Study I: In the manufacturing industry, a jamming attack against an IIoT system describes how the efficiency of operation and data integrity are interfered with. Case Study II: In an IIoT-enabled manufacturing facility, the spoofing attack shows the vulnerabilities associated with identity verification processes, potentially leading to unauthorized access to data and manipulation of operations. These case studies underline the importance of robust security measures to ward off sophisticated cyber threats in industrial settings.

### 4.1. Case Study I: Jamming Attack and Perimeter Breach in an IIoT-Enabled Manufacturing Facility

A jamming attack at a manufacturing facility through IIoT systems halted all production processes and exposed the critical vulnerabilities of the facility’s security infrastructure. The attacker was a former employee who knew the inner workings of the manufacturing facility and discovered gaps in the physical security system by bypassing the access controls into the perimeter and installing a powerful jammer inside the operational area [54]. The high-frequency jammer emitted signals that jammed legitimate communications, effectively breaking the connection between IIoT sensors, automation systems, and the central control system [29]. The attack resulted in an extremely severe breakthrough, with significant communication breakdowns among sensors and operational controls, which affected the production workflows and incurred financial loss.

**Figure 5:**
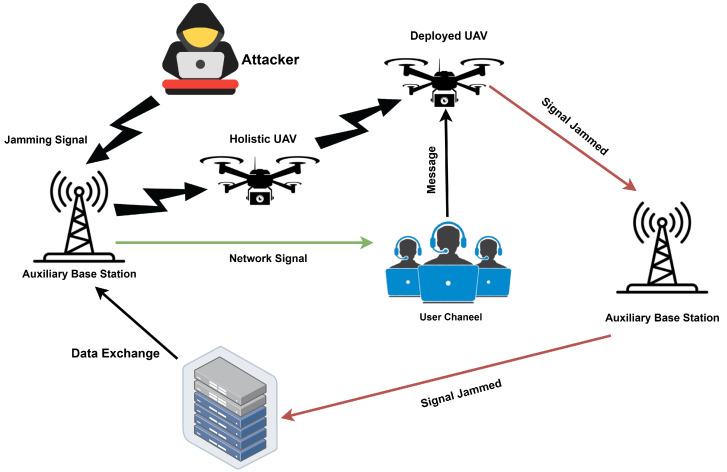
Jamming attack scenario in IIoT.

Figure 5 shows a network communication system under attack by a hacker disrupting or intercepting the signals. In red, the attacker’s actions interfere with the legitimate signal between the servers and the monitoring team, ensuring the system’s integrity. Through green arrows, the monitoring team will receive legitimate signals, working their way to identify and deter malicious activities. This scenario shows how critical good communication security and intrusion detection schemes are for mitigation against jamming and illegal access attacks. It represents a starting point for the study of resilient network security architectures.

- Detailed Effects of Jamming Attacks in IIoT Environments
  – Operational Disruption: The wireless communication gets disrupted, rendering faults in the automated systems and resulting in production jams.
  – Safety Risks: Unscheduled halting on production lines leads to delays in that area and also risks the safety of workers.
- Financial Impact
  – Productivity Loss: Production halts result in lost production and possible contractual fines.
  – Emergency Costs: Costs of problem identification, application of emergency measures, and streaming restorations or replacement.
- Compromised Data Integrity
  – Analytical Errors: Data analytics become erroneous, and inefficiencies are caused by the interruption of data transfers and loss in transfers.
  – Maintenance Challenges: Long-term strategic and maintenance schedules are simulated because of the loss of realism of historical data.
- Mitigation Strategies for Jamming Attacks
  – Dynamic Frequency Hopping: Systems that allow IIoT devices to change frequencies to avoid jamming dynamically.
  – Robust Encryption and Secure Protocols: The wireless signals are encrypted, and secure communication methods protect the data flows.
  – Advanced Detection Systems: Deployment of powerful spectrum analyzers that detect odd frequency activity quickly; frequent security audits and penetration testing to learn and strengthen feebleness.
  – Redundant Communication Pathways: Create multiple communication channels for necessary control signals to ensure the system’s integrity.

#### Mathematical Model of Jamming Attack

In this case study, a disgruntled former employee executed a jamming attack on an IIoT system in a manufacturing plant. The attacker used a high-power jammer to interfere with the frequencies utilized by the plant’s wireless sensors and automation controls, causing significant operational disruption and financial losses. Let $P_{j}$ represent the power of the jamming signal, $P_{s}$ the power of the legitimate signal, and $N_{0}$ the noise power spectral density. The signal-to-interference-plus-noise ratio (SINR) can be modeled as in Equation (1):

(1) $$\mathrm{SIN}R=\frac{P_{s}}{P_{j}+N_{0}}$$

A successful jamming attack occurs when SINR drops below a certain threshold γ. Thus, the condition for jamming can be written as in Equation (2):

(2) $$\frac{P_{s}}{P_{j}+N_{0}}<\gamma$$

The impact of the jamming attack is modeled as follows:

1. Operational Disruption: It may result in production halts. Let $T_{p}$ represent the total production time affected. The operational downtime can be modeled as $D_{o}=\alpha T_{p}$, where α is the percentage of time the jamming attack disrupted operations.
2. Financial Impact: It may result in productivity loss. Let $L_{p}$ represent the loss in productivity per hour. The total productivity loss $L_{t}$ can be computed in Equation (3):
  (3) $$L_{t}=L_{p}\times T_{p}$$
3. Compromised Data Integrity: It may lead to analytical errors. Let ϵ represent the error rate in data analytics due to data corruption. The overall error can be expressed as E=ϵ×D, where *D* is the amount of data affected.
4. Safety Risks: The safety risks are defined as unscheduled halts. Unscheduled halting on production lines leads to delays in that area and risks workers’ safety.

The mitigation strategies can be modeled as follows:

1. Dynamic Frequency Hopping: Frequency hopping can be modeled by dividing the available frequency band *B* into *n* subbands, each of bandwidth Δf. The system switches frequencies periodically to avoid jamming, as represented in Equation (4):
  (4) B=n×Δf
2. Robust Encryption and Secure Protocols: Let $E_{k}$ represent the encryption strength. The probability of successful decryption by an attacker can be modeled as in Equation (5):
  (5) $$P_{d}=\frac{1}{2^{E_{k}}}$$

### 4.2. Case Study II: Spoofing Attack in IIoT-Enabled Manufacturing Facility

RFID was utilized within an industrial plant to track inventories and monitor equipment. This attack demonstrates a legitimate spoofing attack against an RFID system by forging authentication signals to impersonate legitimate devices [8]. It resulted in the manipulation of systems, for instance, triggering a false update of an inventory or interfering with equipment workflows. The spoofing attack exploited a vulnerability in the pipe of data processing of the RFID network. Contrary to what has been presented above, data collected by RFID tags directly did not go to the cloud. Instead, such data were processed at the edge or fog layer to clean, compress, and encrypt data transmitted selectively to the cloud. It is scalable, cost-efficient, and operationally viable.

**Figure 6:**
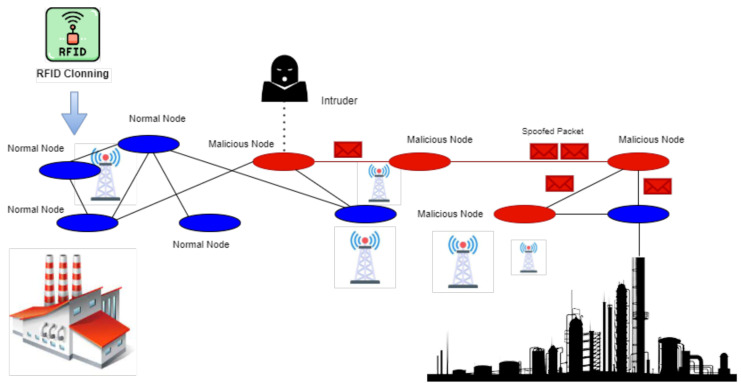
Spoofing attack scenario in IIoT.

Industrial Network Architectures: Typically, several layers are involved, such as sensing, edge computing, and cloud platforms. Most real-time functionality would be at the edge layer, and long-term storage and analytics would be in the cloud. This multi-layered architecture significantly restricts reliance on the significant use of cloud resources for live operations. Figure 6 illustrates an example of a network spoofing attack using RFID cloning. It elaborates on the impact of cloned RFID data in network communications from the different nodes, for instance, blue and red ovals. Data integrity may be compromised at the points of transmission towers and industrial sites, both essential nodes. The picture expresses the utilization of the high-security features that foil and minimize such attacks.

1. Details of the Attack
  - Attack Vector: Advanced techniques were employed to simulate network communication protocols to provide the central control system with false signals.
  - Impact: Unexpected actions by automated machinery cause production errors, unscheduled downtime, and safety issues.
2. Mitigation and Response
  - Immediate Response: Anomalies within system outputs were detected, and the affected devices were separated.
  - Security Upgrades: Implemented robust authentication and encryption protocols for data transmissions.
  - Ongoing Monitoring: Improved network monitoring techniques were implemented to find instances of unwanted access.

#### Mathematical Model of Spoofing Attack

In this case study, an attacker conducted a spoofing attack by sending fake data packets into the network, simulating legitimate communications between sensors and the control system. Let $P_{\mathrm{false}}$ represent the probability of detecting a spoofed signal as legitimate. The total number of spoofed signals $N_{s}$ can be modeled in Equation (6):

(6) $$N_{s}=\sum_{i=1}^{n}S_{i}\times P_{\mathrm{false}}$$

where $S_{i}$ represents the number of spoofed signals at the *i*-th instance.

The impact of the spoofing attack is modeled as follows:

1. Operational Impact: Spoofing attack may result in unexpected actions. Let $F_{m}$ represent the frequency of machine malfunctions. The total number of malfunctions $M_{t}$ due to spoofing can be expressed in Equation (7):
  (7) $$M_{t}=F_{m}\times T_{s}$$
  where $T_{s}$ is the total time the spoofing attack persisted.
2. Safety Risks: It may lead to unscheduled downtime. The downtime $D_{s}$ due to safety risks can be modeled as in Equation (8):
  (8) $$D_{s}=\beta \times T_{s}$$
  where β is the percentage of time operations were halted for safety checks.

The mitigation and response to spoofing attacks can be performed as follows:

1. Robust Authentication: The probability of a successful spoofing attempt $P_{s}$ can be reduced by enhancing authentication mechanisms. If *A* represents the authentication strength in Equation (9), then:
  (9) $$P_{s}=\frac{1}{2^{A}}$$
2. Ongoing Monitoring: Let $M_{d}$ represent the monitoring detection rate. The probability of detecting a spoofed signal $P_{d}$ can be modeled as in Equation (10):
  (10) $$P_{d}=1-{\left(1-M_{d}\right)}^{n}$$
  where *n* is the number of monitoring instances.

## 5. Proposed Architecture

**Figure 7:**
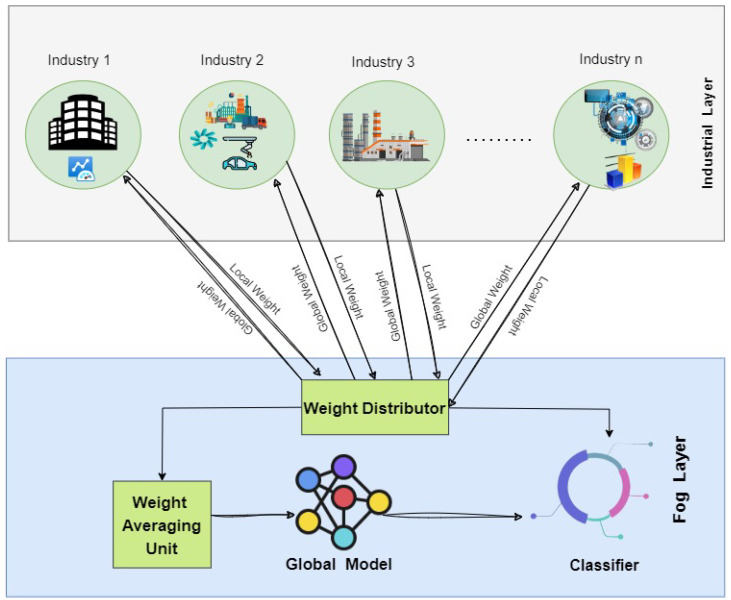
Proposed architecture.

The system architecture is composed mainly of two layers, as shown in Figure 7: the Fog layer and the Industrial layer, both inextricably linked in the core idea of the proposed research. The Fog layer includes critical components that synergize to achieve the research objectives. These components consist of a Weight Averaging Unit, a CNN model, classifiers, and a Classifier Sending Unit responsible for distributing weight information to the Weight Distributor. The proposed design follows a structured process that involves weight determination, subsequent refinement, and the seamless distribution of these weights to the Industrial layer.

This layer is very substantial architecturally and constitutes several distinctive sectors of regional importance. It is unique in its ability to offer localized training customized to the complexities of IIoT organizations. This methodology helps businesses improve and tune their models to operational complexity. The Industry layer adds its refined local weight to the global model positioned in the Fog layer, which is the epitome of such specialized training. The effectiveness of this architecture lies in how these two different layers interplay with each other. The global weights, when integrated, derived from the Fog layer, turn the Industry layer into a more objective and robust prediction level. At the same time, the local weights of the Industry layer add contextually nuanced insights into the whole global model. The smooth integration of these elements underlines the efficiency of the architecture, hence showing that it can guide significant development in the specific domain of the research.

We now provide an explanation of Algorithm 1 for intrusion detection using CNNs. The process starts with the random initialization of weights and biases, w(0) and b(0). Then, for t=1 to *T*, the IIoT data Xi and corresponding labels $y_{i}$ for each smart meter i={1 to *K*} train the convolutional neural network CNNi and update its model weights locally, $w{\left(t\right)}_{i}$. Taking the mean of local weights for K iterations gives Equation (11):

(11) $$\frac{1}{K}\sum_{i=1}^{K}w{\left(t\right)}_{i}$$

which is used to update the global weights, w(t). Similarly, using Equation (12), the mean of bias iterations are calculated and applied to each $CNN_{i}$:

(12) $$\frac{1}{K}\sum_{i=1}^{K}b{\left(t\right)}_{i}$$

The local models, after updates, progress further in each iteration over time. Each smart meter predicts $X_{i}$ for intrusion detection and produces an output, ${\hat{y}}_{i}$. The judgment variable is obtained by comparing the value of ${\hat{y}}_{i}$ with the threshold value. If ${\hat{y}}_{i}$ is greater than or equal to the threshold, it is judged as ‘Intrusion detected’; otherwise, ‘No intrusion’. After T iterations, the algorithm’s output will be the global weights *w* and bias *b*.
**Algorithm 1** Proposed algorithm for intrusion detection  1:Initialization: Randomly initialize weights $w^{\left(0\right)}$ and bias $b^{\left(0\right)}$ for CNN models.  2:**for** t=1 to *T* **do**                 ▹ Federated Learning Iterations  3:    **for** each client i=1 to *K* in parallel **do**  4:        Train local CNN model on dataset $X_{i}$ with labels $y_{i}$.  5:        Compute model updates $w_{i}^{\left(t\right)}$ and $b_{i}^{\left(t\right)}$.  6:    **end for**  7:    Aggregate updates on the global server:
$$w^{\left(t\right)}=\frac{1}{K}\sum_{i=1}^{K}w_{i}^{\left(t\right)},\;b^{\left(t\right)}=\frac{1}{K}\sum_{i=1}^{K}b_{i}^{\left(t\right)}.$$  8:    Update local CNN models with global parameters.  9:**end for**10:**for** each client i=1 to *K* **do**11:    Perform intrusion detection: ${\hat{y}}_{i}={\mathrm{CNN}}_{i}\left(X_{i}\right)$.12:    **if** ${\hat{y}}_{i}\geq \mathrm{Threshold}$ **then**13:        Intrusion Detected.14:    **else**15:        No Intrusion.16:    **end if**17:**end for**

## 6. Proposed Methodology

**Figure 8:**
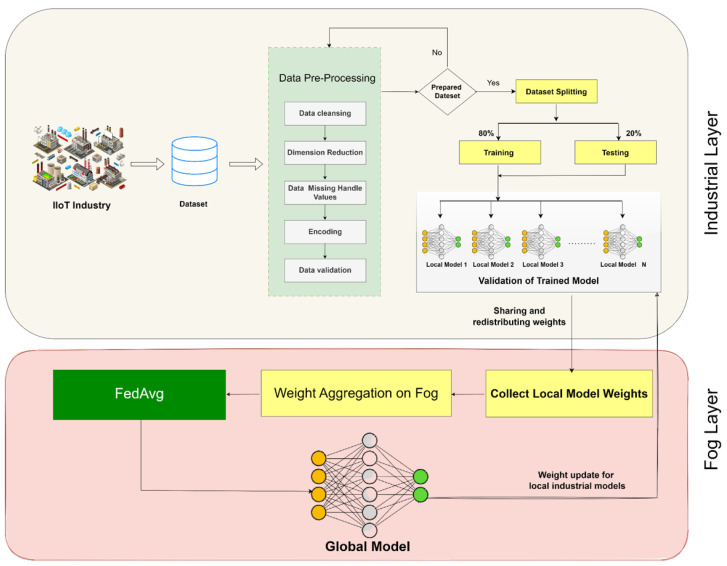
Proposed model for intrusion detection in IIoT environments.

The proposed methodology, as depicted in Figure 8, represents a Fog-enabled Federated Learning-based Intrusion Detection System, namely FFL-IDS. The system, so developed, is anticipated to counter critical security threats such as spoofing and jamming attacks on the IIoT application while maintaining scalability, low latency, and robust intrusion detection. The integration of fog computing with federated learning ensures data privacy as well as decentralized intrusion detection.

### 6.1. Key Features of the Proposed Model

1. Decentralized Local CNN Models: Every node at the fog layer hosts a localized CNN, which is learned on its dataset to learn patterns specific to the environments. The parameters of such local models are aggregated by employing the fedAvg algorithm and achieve a global model without requiring raw data sharing.
2. Privacy Preservation: The raw data are stored in IoT devices; henceforth, the data do not flow toward the fog or cloud layers. To be included in the model updates that will be averaged, it adds Gaussian noise.
3. Fog Layer as the Processing Hub: The fog layer preprocesses data, facilitates local model training, and aggregates model updates. It reduces latency and supports real-time intrusion detection.
4. Global Model Refinement: The global server collects updates from all nodes, sharpens the global model using privacy-preserving techniques, and then redistributes the sharpened model for improvements.
5. Scalability and Robustness: It is scalable over multiple nodes and shows resilience under large-scale IIoT attacks. Experimental results using the Edge-IIoTset and CIC-IDS2017 datasets confirm its effectiveness with high-performance metrics.

### 6.2. Workflow Description

1. Data Collection and Preprocessing: IoT devices generate raw data and perform basic preprocessing locally, such as normalization and feature extraction, to reduce transmission overhead.
2. Local Model Training: Each fog node trains a CNN model using its local dataset. The CNN architecture includes:
  - Conv2D Layers: Extract spatial features using convolution filters.
  - MaxPooling2D Layers: Reduce dimensionality and enhance computational efficiency.
  - Dense Layers: Map extracted features to intrusion predictions.

The model is optimized using the Adam optimizer to minimize the loss function.

1. Privacy-Preserving Model Aggregation: The local models broadcast their updated weights and biases to the global server. In the name of privacy, Gaussian noise is added to those updates. The FedAvg algorithm aggregates those updates to update the global model.
2. Global Model Distribution: The polished global model is returned to all the fog nodes, so constant learning and intrusion pattern adaptation can occur.
3. Intrusion Detection: Each fog node utilizes the evolved global model to evaluate its local IIoT data in real time and checks them against possible intrusions that have been set based on predefined thresholds.

### 6.3. Proposed Algorithm: Federated Learning for Intrusion Detection

Algorithm 1 demonstrates the complex intrusion detection procedure in the IIoT, using federated learning with CNN. It describes the pseudocode in a step-by-step manner: initialization of model parameters, iterative training across local devices, model updates aggregation at the global server, and the decision-making process of intrusion identification. It ensures privacy preservation while scaling to extend its versatility to achieve real-time responsiveness.

### 6.4. Enhanced Description of Figure 8

Figure 8 illustrates the architecture of the proposed FFL-IDS framework. It consists of:

- IoT Devices: Responsible for generating raw data and performing basic preprocessing to reduce the computational load.
- Fog Layer: Acts as a processing hub, facilitating local training, model aggregation, and real-time threat detection. It coordinates the collaborative training process while preserving privacy.
- Global Server: Aggregates model updates from multiple fog nodes using FedAvg and redistributes the refined model to improve intrusion detection capabilities across all nodes.

The figure visually captures the iterative process of federated learning, demonstrating how local models are continuously refined to detect intrusions with high accuracy while maintaining data privacy.

## 7. Dataset Preparation and Analysis

This section presents the dataset and details the feature extraction and preprocessing.

### 7.1. Dataset Preparation

We first imported the Edge-IIoTset and CIC-IDS2017 datasets into pandas DataFrames for our proposed FL model to enable data processing. These include the preprocessing procedure of filling in the missing values, standardizing numerical characteristics to a scale, and encoding approaches to transform categorical data. Then, we carry out a feature examination to understand the dataset’s attributes in greater detail. Afterwards, we divide the datasets into separate subsets describing different clients in the FL framework. Subsets are distributed over multiple nodes, mimicking the data distribution in a federated network setting. It ensures the model is resilient in jamming and spoofing attacks while protecting the user’s privacy.

### 7.2. Data Preprocessing

This study requires many preprocessing activities to optimize data utility. First, we encode the categorical features of the two data sets using one-hot encoding: protocol type, service, and IP addresses. It includes converting these features to numerical values and creating binary variables for each category. This transformation is critical for ML algorithms to analyze data efficiently. Labeling records is a significant aspect: every record has a binary rank, with ‘0’ indicating ordinary traffic and ‘1’ indicating assault traffic. Labeling is critical for binary classification models, allowing the algorithm to distinguish between normal activities and dangerous security breaches. This category classifies the type of attack, such as DDoS, SQL injection, or malware, among others. Multi-class classification methods are essential because they can detect an assault while accurately determining its category. A granular approach is required when installing accurate security measures in NIDS.

### 7.3. Dimension Reduction

Dimension reduction employs Principal Component Analysis (PCA) and feature selection algorithms. PCA transforms the data into a set of linearly independent variables, capturing the most variation with the fewest components. Feature selection approaches, such as mutual data collection and chi-squared tests, discover and preserve traits directly relevant to identifying jamming and spoofing assaults. These include network-specific information, destination and source ports, protocol types, TTL (Time to Live), TCP flags, payload content, connection length, traffic counts, flow statistics, and trends. This scientific technique ensures a focused analysis, reducing superfluous data and increasing the efficacy of the intrusion detection process.

### 7.4. Encoding

The Edge-IIoTset and CIC-IDS2017 datasets use encoding to turn categorical data into numerical representations, which is crucial for machine-learning approaches. Python’s pandas.get_dummies() function uses one-hot encoding to generate binary columns for each category. $x_{i}=1$ shows the presence of category *i*, whereas 0 indicates its absence. Another encoding method, label encoding, is implemented by the ‘sklearn.preprocessing.LabelEncoder’ module, which assigns a unique number to each category. These methods enhance categorical data analysis and preservation of IIoT ecosystem properties.

### 7.5. Data Missing Values Handling

The proposed research deals with issues arising from missing values through statistical imputation taken by the Edge-IIoTset and CIC-IDS2017 datasets. We used mean imputation, as shown in Equation (13):

(13) $$IV=\frac{1}{N}\sum_{i=1}^{N}x_{i}$$

where IV represents the Imputed Value, $x_{i}$ represents the observed values, and *N* signifies the count. For skewed datasets, we go with median imputation, where the imputed value of the data points is the average of the data points {$x_{1}$, $x_{2}$, …, $x_{N}$}. These solutions provide a low possibility of bias and preserve the integrity of the dataset, which is critical for accurate intrusion detection models.

### 7.6. Data Reduction

Various imputation techniques and statistics aim to fill in the missing values while ensuring data integrity. The general mathematical expression used refers to Equation (14), where $x_{i}$ represents the observed values and *N* is the number of items. For data points not generally distributed, median imputation is used; this involves the computation of the median value of the respective data points as {$x_{1},x_{2},\ldots ,x_{N}$}. Furthermore, missing values in categorical data are replaced through mode imputation. The imputed value is determined by computing the mode of the respective classifications represented by {$x_{1},x_{2},\ldots ,x_{N}$}. Through the use of regression imputation in calculating missing values with other variables, it is possible to see the imputation value being defined through Equation (14):

(14) $$\mathrm{Imputed}\;\mathrm{Value}=\beta_{0}+\beta_{1}x_{1}+\beta_{2}x_{2}+\ldots +\beta_{n}x_{n}$$

These solutions reduce the introduction of bias while ensuring that the dataset is reliable for the intrusion detection models.

### 7.7. Data Splitting

We have divided the data methodically to evaluate the models better for the proposed research of the Edge-IIoTset and CIC-IDS2017 datasets. In total, 70% of the dataset was assigned to training, 20% to testing, and 10% to the model validation. This section gives an exhaustive evaluation of the ability of the proposed IDS to detect attacks, including jamming and spoofing within the IIoT environment. Besides, we have enforced an FL system comprising ten unique models that enhance the adjustment of parameters over different subsets of data. Such a technique improves the accuracy and performance of the model, including data safety, which is an essential component of FL.

#### CNN Components and Its Values

The approach of training a CNN model includes several steps. The basic components of the CNN model were as follows:

- Activation Functions: The Rectified Linear Unit (ReLU) function, Equation (15), introduces non-linearity, enabling the model to acquire intricate patterns:
  (15) ReLU(x)=max(0,x),
  The softmax function is employed in the final layer for multi-class classification, denoted in Equation (16):
  (16) $$\sigma {\left(z\right)}_{i}=\frac{e^{z_{i}}}{\sum_{j=1}^{K}e^{z_{j}}},$$
- Pooling Layers: These layers decrease the spatial dimensions of the input volume in preparation for the subsequent convolutional layer. One commonly used technique is MaxPooling, which selects the highest value within a defined window size.
- Fully Connected Layers: These layers, where each neuron is connected to every neuron in the previous layer, calculate the class scores, resulting in a certain volume size, represented in Equation (17):
  (17) O(1×1×numberofclasses).
  They are commonly placed after many convolutional and pooling layers.
- Dropout Technique: This technique combats overfitting by randomly deactivating a proportion of neurons, determined by a dropout rate *p*, during training.
- Optimization Algorithm: Adam, an optimization algorithm, adjusts network weights based on training data iteratively. It combines the Adaptive Gradient Algorithm (AdaGrad) and Root Mean Square Propagation (RMSProp).
- Loss Function: Often, Cross-Entropy loss is used for classification tasks, calculated as represented in Equation (18):
  (18) $$\mathrm{Loss}=-\sum y\log(\hat{y})$$
  where *y* is the actual label and $\hat{y}$ is the predicted probability.

### 7.8. Weights Sharing and Fine-Tuning

The proposed FL strategy comprises training ten local CNN models, labeled as $M_{i}$, where i={1,2,…,10}, using the Edge-IIoTset and CIC-IDS2017 datasets. Every individual model $M_{i}$ is trained on a unique subset of data, and its weights $W_{i}$ are updated during the training process. The central server computes the aggregate of these weights using the formula presented in Equation (19):

(19) $$W_{\mathrm{global}}=\frac{1}{10}\sum_{i=1}^{10}W_{i}$$

Equation (19) calculates the average of the weights from all local models. After the process of aggregation, the global model is subjected to fine-tuning. This stage may involve doing extra training iterations using a specific learning rate η to optimize the global model by leveraging pooled information. The model’s ultimate performance is assessed quantitatively through accuracy, precision, and recall metrics. It guarantees that the model is efficiently trained for intrusion detection in the Edge-IIoTset network.

## 8. Implementation Details

The setup for the experiments that were used to test and implement the proposed model is discussed in this section. It also talks about the platform and tools that were used for implementation. Python 3.7 is the computer language used in our FL-based IDS experiments that use Google Colaboratory. Data preprocessing is made more accessible by Pandas, Scikit-learn, and Numpy, making it easier to change, analyze, and scale the dataset. Some of the hardware used in the trial setup at Google Colab were the following: Google Colab’s RAM size varied depending on the type of runtime chosen. For the high-RAM runtime, it was as much as 12 GB. The CPU tools have multiple cores, simultaneously letting model inference and training happen. NVIDIA GPU acceleration, which can get through Google 60 Colab, works best for DL tasks. TensorFlow Federated (TFF) sets up a FL framework. Its partitioning features build 10 local datasets representing Edge IIoT nodes. CNNs train local models on each local dataset. Through Federated Averaging, the weights and slopes of the local models are added together to make the global model. The GPU support in Google Colaboratory speeds up training, and the efficient model consolidation keeps memory usage low. The experimental setup is further elaborated in Table 3.

### 8.1. Dataset Description

This study aims to evaluate the performance of a distributed IDS using two popular datasets: Edge-IIoTset and CIC-IDS2017. Edge-IIoTset is a large dataset for IoT and IIoT technologies that can be applied to machine-learning-based IDS. A state-of-the-art IoT/IIoT testbed produces this dataset. Hence, it includes data from different types of IoT devices and sensors. The dataset consists of fourteen IoT and IoT connectivity protocol attacks, categorized into five groups: DoS/DDoS, data gathering, man-in-the-middle, injections, and malware attacks. This dataset is remarkable for extracting 61 highly correlated characteristics from the set of 1176 identified features, which makes it applicable to centralized and FL applications in cybersecurity research. The CIC-IDS2017 dataset enhances this by including a wide range of network behaviors and intrusion situations, offering more than 80 network flow variables. Both datasets are selected based on their authenticity and comprehensive categorization, which are essential for developing a highly efficient IDS in the IIoT environment. This dataset was chosen because it has a real testbed, real attack traffic, fake IIoT traffic in an industrial setting, and data that have been carefully labeled.

### 8.2. Description of the Edge-IIoTset Dataset

The intrusion detection system utilizes this cybersecurity dataset for IoT and IIoT applications. This dataset provides the research and classification of 14 different forms of attacks against IoT and IIoT protocols. These attacks fall under five categories of danger: DoS and DDoS, data gathering, injection, man-in-the-middle, and malware attacks. Among the 1176 features, 61 depict a high correlation. The dataset comprises 20,952,648 attack facts, of which 11,223,940 are expected, and the remaining 9,728,708 represent the number 27 attacks. The dataset was divided into 20% testing and 80% training. The stratification option ensured the consistency of the percentages across classes. The dataset represented 1,909,671 samples, of which 1,527,736 belonged to the training set and 381,935 formed the test set. These samples were arranged into fifteen classes. Table 4 provides an overview of the Edge-IIoTset dataset traffic types and their counts. The table divides network usage between benign traffic and different kinds of cyber-attacks and summarizes the extent of the dataset.

### 8.3. Description of the CIC-IDS2017 Dataset

The dataset has been partitioned into 15 categories, with 14 categories representing different network attack methods and 1 category representing benign traffic. Of all the recorded events, 2,273,097 can be classified as benign traffic, while 557,646 cases belong to the category abnormal. The CIC-IDS2017 dataset is a subset of the Intrusion Detection Evaluation Dataset. It comprises 85 data features and contains five days of data starting on approximately 3 July 2017 and ending on 7 July 2017. The attacks include Botnets, Distributed Denial of Service (DDoS) incidents, Web Attacks, DoS, Brute Force Intrusion, Brute Force FTP, and Force SSH attacks. Table 5 summarizes the detailed characteristics of the CIC-IDS2017 dataset.

## 9. Evaluation and Results

The simulation results are rigorously investigated to provide relevant insights into the usefulness of the proposed intrusion detection architecture using the Edge-IIoTset and CIC-IDS2017 datasets. These performance parameters are routinely compared to recognized benchmarks or current intrusion detection methods. This comparison analysis, a cornerstone of our research, is critical for determining the effectiveness and operational efficiency of the proposed system. Furthermore, such evaluations assist in identifying areas where the proposed model outperforms previous approaches, highlighting its potential contributions to advancing network security.

### 9.1. Accuracy

**Figure 9:**
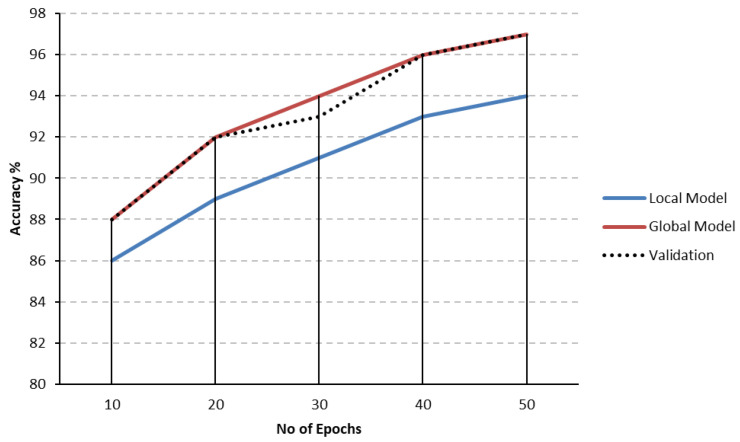
Accuracy of test models and Edge-IIoTset validation results.

In our experiments, accuracy is defined as the model’s capacity to correctly identify or forecast the results of the given data samples (i.e., incursions). The calculation involves dividing the total number of intrusions handled by the model by the number of accurately recognized intrusions. This measure shows how well the model accurately recognizes the target variable or class labels, like an incursion. Over 50 epochs of FL, the accuracy of validation sets and local and global models for the Edge-IIoTset dataset steadily rises. The global model begins with an accuracy of 88%, while the local model begins with 86% accuracy. The validation accuracy closely resembles these numbers, at about 87%. Both models demonstrate notable improvements throughout training; by the 50th epoch, the global model has reached 97%, and the local model has reached 94%, with validation accuracy reaching a peak of 96.4%. Figure 9, which shows the accuracy patterns for test and validation sets, presents these findings.

**Figure 10:**
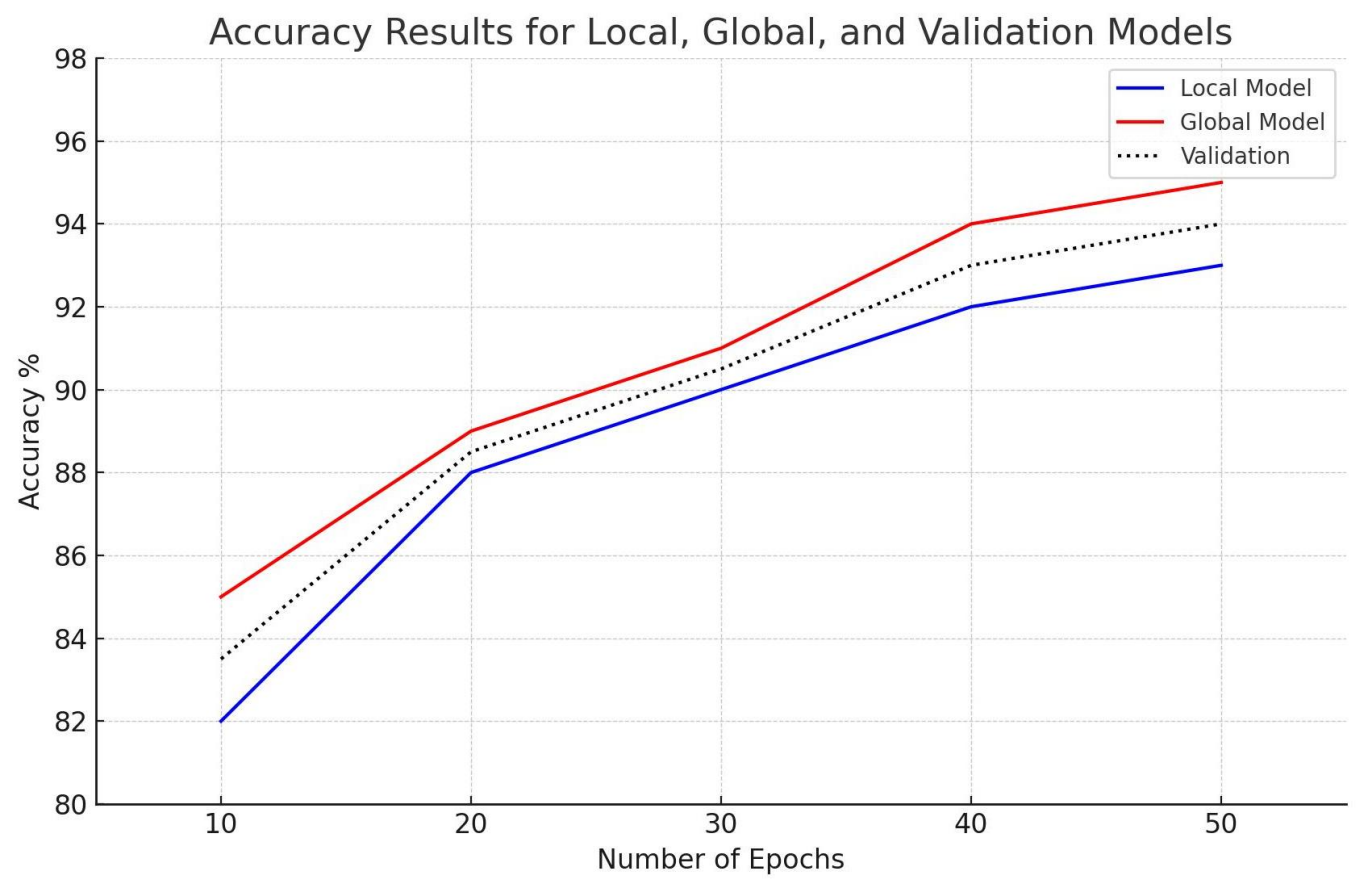
Accuracy of test models and validation results for CIC-IDS2017.

Comparably, in Figure 10, for the CIC-IDS2017 dataset, the initial validation accuracy is 83.9%, and the accuracy starts at 82% for the local model and 85% for the global model. By the end of the 50 epochs, the local and global models’ accuracy stabilizes at 93% and 95.8%, respectively, with 94% being the validation accuracy. The patterns depicted in Figure 10 demonstrate a visible and steady rise in test and validation accuracies, indicating that the FL model converges to dependable performance levels. Empirical findings from both datasets demonstrate that allowing models to go through lengthy training times significantly improves the predicted accuracy of the models.

The FL model’s ability to adapt to local data adjustments specific to each dataset is evident in the consistent performance improvement across both datasets. The accuracy values for the test and validation sets, showing strong similarity with only nominal differences between datasets, are a testament to the high generalization ability of the model. The near-perfect correspondence between the test and validation accuracy for the CIC-IDS2017 dataset further strengthens the model’s performance on unseen data. These results provide a strong indication of the potential of FL in remote data sources, especially where data privacy is a concern. This approach proposes an ideal balance between the protection of sensitive data and the enhancement of model performance. The privacy-preserving nature of FL makes it a promising solution for data privacy during model training.

### 9.2. Precision

An essential measure for considering a model’s good prediction is precision. It is the proportion of all optimistic predictions that are true—the number of true positive instances correctly identified divided by all positive predictions (including false positives, which are negative instances misclassified). Because of the dire repercussions of incorrect classifications, this statistic is essential in situations such as IDS.

**Figure 11:**
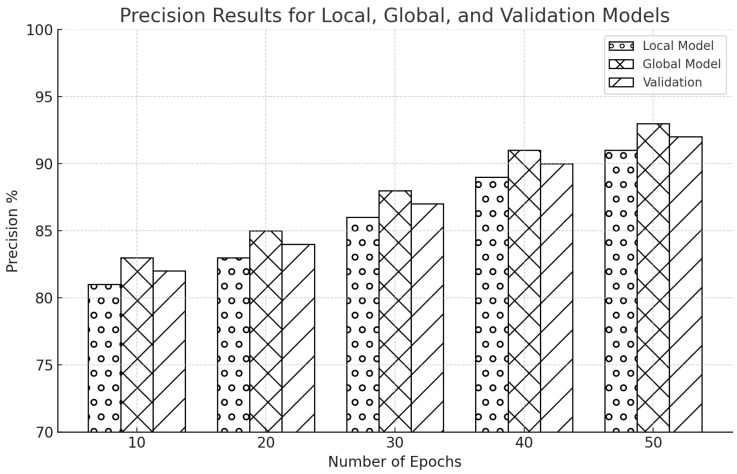
Precision of local and global models and validation results for Edge-IIoTset.

Figure 11 shows a steady increase in model precision over time. The global model’s precision was higher at 84%, while the local model’s initial precision was 82%. The validation results were close to 83%. The local model’s precision increased to 86%, the global model to 88%, and the validation to 87% as the training went on to 20 epochs. The local model was at 88% precision by the 30th epoch, the global model was at 90%, and the validation findings were at 89%. By the 40th epoch, the trend was still rising, with the local model reaching 91% precision, the global model at 93%, and validation at 92%. Precision peaked after 50 epochs, with the global model at 95%, the local model at 93%, and validation results indicating a substantial 94%.

**Figure 12:**
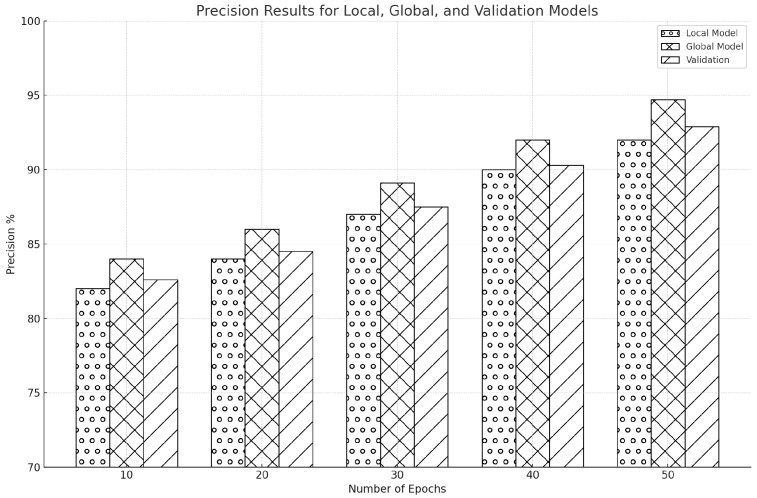
CICIDS-2017: precision of test models and validation results.

There is a similar improvement in precision for the CIC-IDS2017 dataset, as shown in Figure 12. With a validation rate of 83.2% and an estimated 82% precision for the local model, these models were likely to rise over the epochs consistently. The precision was expected to increase to 84% for the local model, 86% for the global model, and 85% for the validation by the 20th epoch. As we proceeded to the 30th epoch, we anticipated that the precision would rise to 87% for the local model, 89% for the global model, and 88% for the validation. We expected more improvements as the models got closer to the 40th epoch, with the validation model hitting 91%, the global model hitting 92%, and the local model hitting 90% precision. The prediction was that the precision would peak at 92% for the local model, 94.9% for the global model, and roughly 93% for validation by the last 50th epoch.

It vividly reveals the global and local models’ accuracy across multiple epochs. The visualizations further clarify that, in our work, the global model outperforms the local model in each iteration. This consistent superiority of the global model in our research emphasizes the power of FL in enhancing the accuracy of network intrusion detection, hence reducing the chances of jamming and spoofing attacks. Detailed analyses of precision over epochs for both datasets provide robust evidence on how FL can improve the prediction precision of IDS. A steady and significant increase in precision across successive epochs shows the increasing ability of models to filter out false positives—a vital pointer to the resilience of cybersecurity measures against dynamic network environments.

### 9.3. Recall

**Figure 13:**
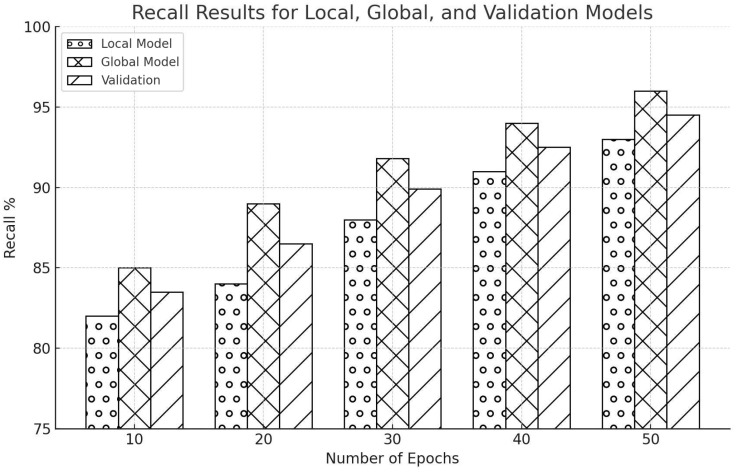
Recall of local and global models and validation results for Edge-IIoTset.

Recall, mostly known as sensitivity or true positive rate, refers to a model’s ability to identify all positive events from a given dataset correctly. The initial results with the Edge-IIoTset dataset were excellent; the local model showed a recall rate of 82%, the global model started with 85%, and the average recall rate was 84.7%. Figure 13 brought out the potential of the FL methodology in improving the accuracy of global models. By the 20th epoch, it had leaped significantly: the recall of the local model surged to 84%, and the global model recall increased manifold to 89%, making for an average of 87.4%. These results demonstrate that FL can effectively amalgamate information from distributed local models, enhancing overall system performance. During the training process, after the 30th epoch, the recall of the local model reached 88%, while the recall of the global model was 91.8%. It indicates consistent improvement and demonstrates the efficiency of FL in boosting the model’s accuracy. At the 40th epoch, the local model achieved a recall rate of 91%, while the global model achieved a recall rate of 94%. This phase emphasized the advantages of cooperative learning among models in a federated environment. After 50 epochs, both models achieved strong recall rates. The local model had a recall rate of 93%, while the global model had a recall rate of 96%. The validation recall rate was likewise excellent at 94%. This experiment confirmed that FL constantly prioritizes the global model over the local one, confirming its effectiveness.

**Figure 14:**
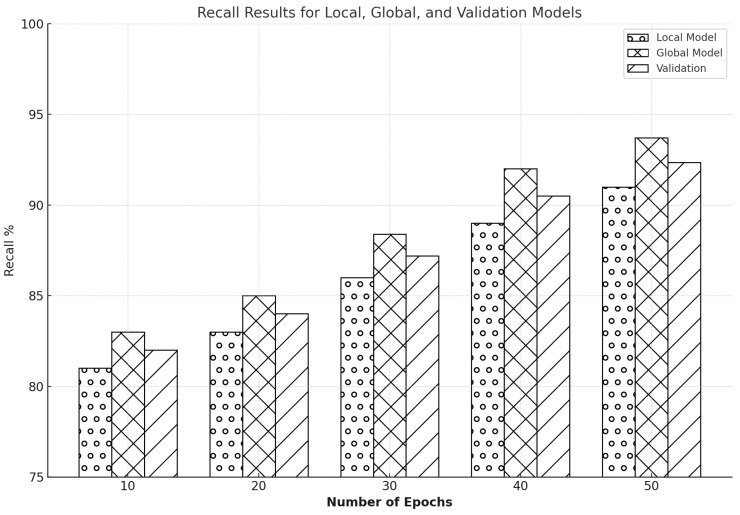
Recall of test models and validation results for CIC-IDS2017.

The recall results exhibited a consistent pattern of gradual enhancements over successive epochs, as illustrated in Figure 14. After ten epochs, the local model initially got a recall rate of 81.7%, while the global model started elevation at about 83.4%. As it went through epochs, the values of recall improved incrementally. By the 20th epoch, the local model achieved an estimated recall of 81.7%, and the global model achieved approximately 85%. By the 30th epoch, the local model could test an accuracy of around 86%, and the international model reached 88% accuracy. By the 40th epoch, the recall values for both the local and global models increased to 89% and 92.7%, respectively. After 50 training epochs, the regional model achieved a recall rate of 91%.

The results underline the strong performance of FL in enhancing recall on both datasets. Our study’s findings indicate that integrating FL with a CNN model can significantly elevate IDS’s precision within the industrial IoT context, subject to jamming and spoofing. We can harden the connectivity security between various IIoT network devices with FL. It is a big step forward in detecting and preventing threats, all while ensuring users’ privacy. The significant improvement of the starting point of 85% to the final recall rate of 96% in the global model clearly shows the significant impact of FL on network IDS, making us feel optimistic about the potential implications of our study.

### 9.4. F1 Score

**Figure 15:**
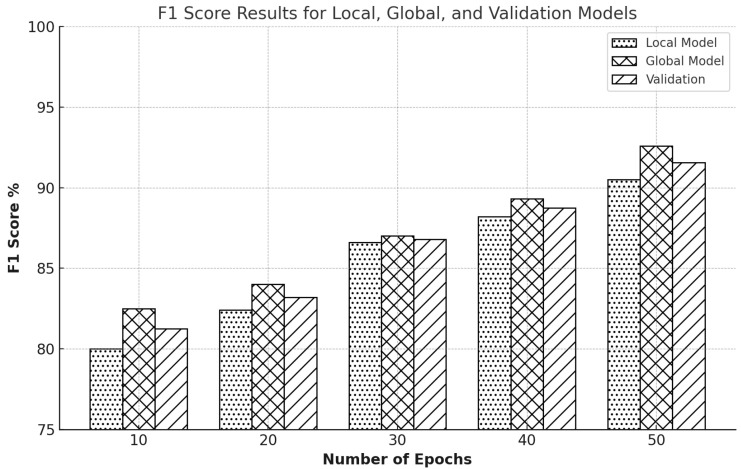
F1 score of local and global models and validation results for Edge-IIoTset.

F1 score is one of the most critical statistics in assessing balance in precision and recall, giving information about the harmonic mean of these two performance quantities. This statistic is vigorous in evaluating IDS’s effectiveness, as both precise identification of real positives and avoidance of false negatives are required. Figure 15 shows an initial F1 score of 80% for the local model and a slightly improved 82.5% for the global model after ten epochs, and the F1 scores continuously improve as the epochs reach 20. The improvement indicates that the models have improved their balance between precision and recall. Specifically, the 20th epoch saw the F1 scores for the local model go up to 82.4%, the global model up to 84.4%, and validation at 83.4%. Subsequently, at the 30th epoch, the F1 scores for the local model were up to 86.6%, while those for the global model were up to 87%. It was a trend until it reached 88.2% for the local model and 89.3% for the global model at the 40th epoch. After 50 epochs, both models’ highest F1 scores were reached; the local model had improved to 90.5%, while the global model had increased to 92.6% with validation at 91.5%. This transition shows that the models can detect better as time goes on. FL effectively improves the balance of precision and recall as time progresses.

**Figure 16:**
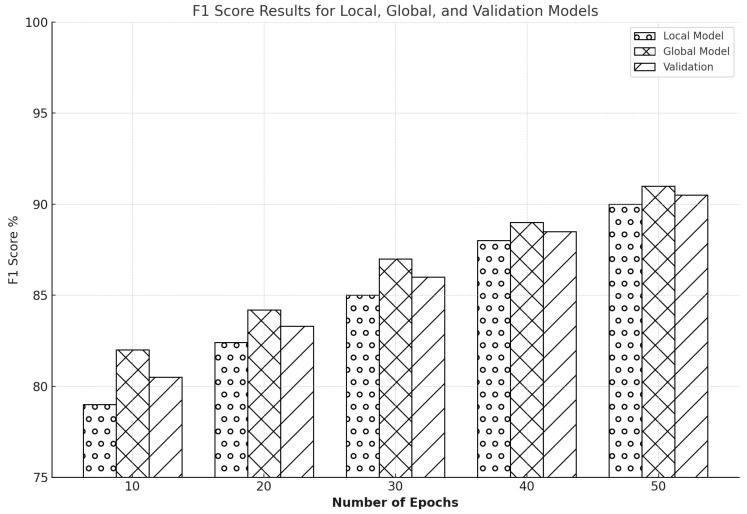
F1 score of local and global models and validation results for CIC-IDS2017.

The graph-based results for the CIC-IDS2017 dataset show an equivalent trajectory of improvement in Figure 16. At ten epochs, the local model has an F1 score of roughly 80%, but the global model has a slightly higher start score of around 83% with validation across 82.1%. As the epochs progress, the F1 scores increase noticeably; by the 20th epoch, the local model has gained approximately 83%, and the global model has reached 84.8%. The 30th epoch shows the local model at approximately 86.4% and the global model at 87.3%. By the 40th epoch, the local model had an F1 score of around 88%, while the global model was slightly ahead at 89.9%. The training concludes at the 50th epoch, with the local model achieving an F1 score of 91% and the global model achieving an impressive 93% with a validation of 91.8%.

The bar graph shows that the global model always performs better than the local model across all epochs, which shows FL’s effectiveness in optimizing NIDS. This increment in F1 scores across the successive training phases reflects that FL enhances the precision and recall of the model and achieves well-balanced performance, which is necessary for effectively detecting and mitigating jamming and spoofing attacks and guaranteeing privacy for network users. The study has shown that FL helps enhance the capability of network IDS; hence, FL is one of the essential methods in cybersecurity.

### 9.5. Specificity

Specificity, a crucial parameter for IDS, assesses a model’s capacity to correctly identify true negatives, preventing non-intrusive actions from being mistakenly categorized as threats. The integrity and effectiveness of network security solutions depend on this parameter. Across several training epochs, we have examined specificity for the Edge-IIoTset and CIC-IDS2017 datasets. The Edge-IIoTset dataset collects real-world data from industrial IoT devices, while the CIC-IDS2017 dataset is a benchmark dataset widely used in intrusion detection. We used FL to improve model performance on these datasets.

**Figure 17:**
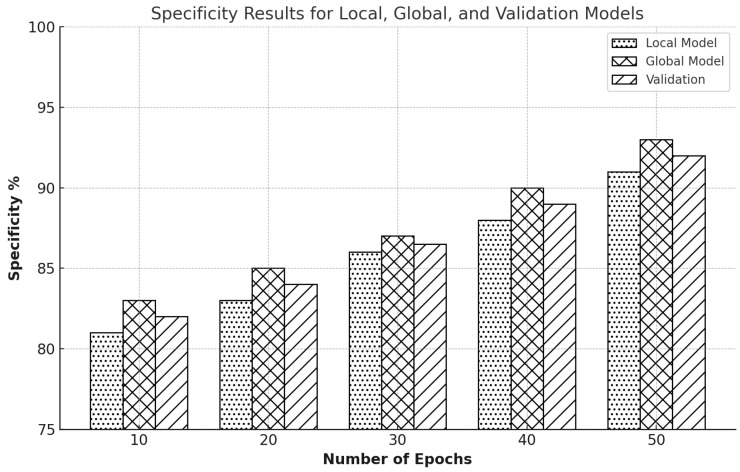
Specificity score for models and validation results for Edge-IIoTset.

The local model for the Edge-IIoTset dataset had a specificity of 81% during the first ten epochs of training. In contrast, the global model had a slightly higher specificity of 83% with 82.4% validation as in Figure 17. This suggests that the global model can more accurately discard non-intrusive behaviors from the outset. Improvements were visible by the 20th epoch when the global model was 85%, and the local model was 83% with 84.3% validation. The specificity of the global model reached 87% and that of the local model, 86% by the 30th epoch. During the 40th epoch, this upward tendency persisted, with the global model attaining 90% specificity and the local model reaching 88% with an average of 89.2% for the validation. The local model achieved a specificity of 91%, and the global model achieved 93% in the final measure after 50 epochs, indicating notable long-term improvements in reliably identifying non-intrusions.

**Figure 18:**
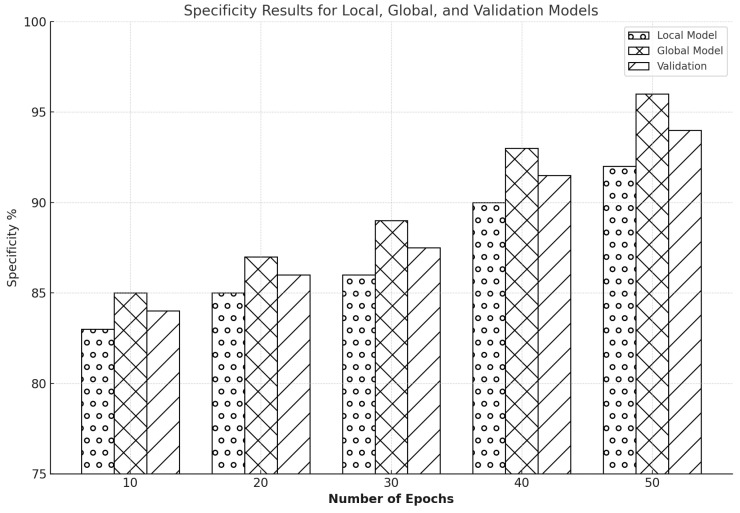
Specificity score for models and validation results for CIC-IDS2017.

As shown in Figure 18, after ten epochs, the specificity of the global model in the CIC-IDS2017 dataset reached 85%, while the local model started at a specificity of about 83%, with validation at 84.2%. Both models showed gradual improvements as training continued; by the 20th epoch, the global model had 87% specificity and the local model approximately 85% specificity with validation at 86.2%. The specificity of the global model increased to almost 89% by the 30th epoch, while the specificity of the local model reached about 86.2%. The global model had a specificity of 93%, and the local model had a specificity of about 90% at the 40th epoch with a validation of 91.4%. The local model achieved a specificity of approximately 92%, and the global model peaked at 96% by the conclusion of the 50 epochs, demonstrating strong performance in the precise classification of non-intrusive behaviors.

A recurring feature emerges from evaluating the two datasets: in terms of specificity, the global model consistently performs better than the local model. It shows how FL, a distributed learning approach that allows multiple parties to collaboratively train a model without sharing their data, can improve the precision of network IDS. FL dramatically enhances the models’ capacity to reject false positives, which is essential for cutting down on pointless alarms in network security operations, in addition to improving the models’ ability to identify real positives by utilizing data from several dispersed sources.

These results highlight the significant benefits of using FL techniques to increase IDS’s specificity. FL contributes to network integrity maintenance and ensures the effectiveness and efficiency of security systems by improving the accuracy of identifying non-intrusions.

### 9.6. Comparison with the State-of-the-Art Model

The proposed model was compared with the lightweight Stacking Ensemble Learning (SEL) model to demonstrate its effectiveness and efficacy [55]. Table 6 shows a comparative analysis of performance metrics between the base model (SEL) and the proposed model, detailing improvements across accuracy, recall, precision, and F1 score. The results reveal that the proposed model outperforms the SEL model on all measures assessed. Firstly, the proposed model achieved 97% accuracy compared to the base model’s accuracy of 87.37%. Secondly, it achieved a recall rate of 96%, compared to the SEL model’s rate of 89.92%. Because our model has a higher recall, it can detect positive cases and network intrusions more precisely than the SEL model. Thirdly, the proposed model achieved a precision of 93%, whereas the SEL model achieved a precision of 90.65%. Accurately detecting affirmative situations is an essential aspect of precision, and the model’s higher precision demonstrates its accuracy in categorizing invasions. Finally, the F1 score, which combines precision and recall, was 92.6% for our proposed model, as opposed to 86.90% for the SEL model. The proposed model’s balance of precision and recall produces consistent results and it regularly surpasses the SEL model.

The CICIDS-2017 dataset was used in our research to assess the proposed model, and accuracy, precision, and recall were compared to the findings of a base paper [56]. At 95.8%, our model’s accuracy was 7.1% higher than the basis paper’s 89.23%, indicating a significant improvement. This improvement suggests better network traffic detection and classification, which is essential for reducing false positives. Additionally, precision increased to 94.9%, up from 92.23% by 2.67%, indicating improved accurate identification. Furthermore, recall increased by 4.58% to 94.5% from 89.92% in the base article, demonstrating the higher proportion of actual intrusions that our algorithm can detect. These enhancements, made possible by our model’s sophisticated CNN architecture, highlight how much better our model is at detecting network intrusions and strengthening security protocols in systems that use the CIC-IDS2017 dataset.

### 9.7. Confusion Matrix

**Figure 19:**
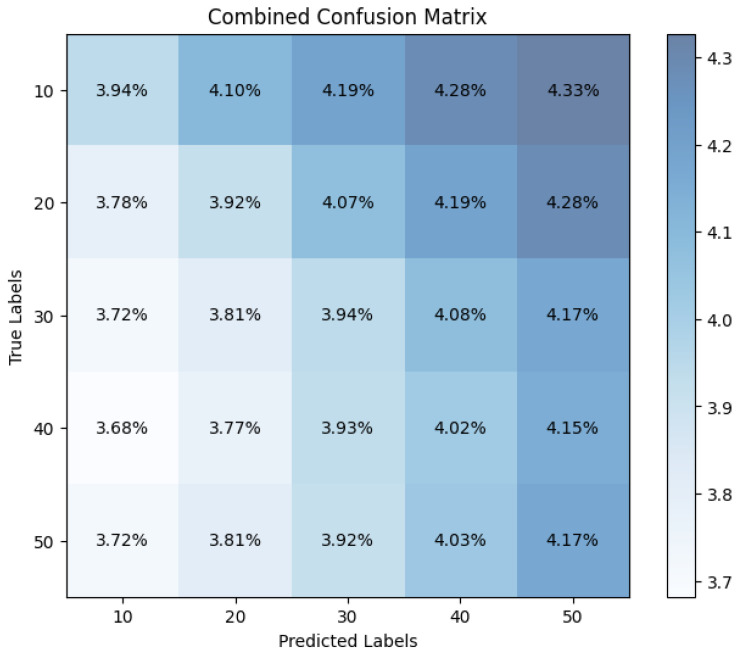
Confusion matrix.

The proposed research, Privacy-Preserved NIDS, indicated that mitigating jamming and spoofing attacks in IIoT contexts requires using a confusion matrix illustrated in Figure 19. The Global Model’s recall rate of 91.16% means fewer false negatives, proving its ability to classify favorable circumstances. In the framework of the IIoT environment, where risk detection is a critical issue, the Global Model’s ability to purge false negatives is crucial. There is higher specificity for the Global Model, which can identify adverse events more precisely. The Global Model has a more remarkable ability to predict which cases are negative more accurately, with a specificity rate of 87.08%. In comparison, the Local Model only shows an 85.54% rate of correctly classifying negative cases. The above-mentioned skill is crucial when reducing false positives is most important. The results of this study provide evidence for the improved performance of the Global Model across several parameters and highlight its practical benefits in the IIoT. The proposed study uses an analytic approach to evaluate the performance of the privacy-preserved NIDS and devise strategies to fine-tune the detection to increase accuracy and reduce false positives.

## 10. Challenges and Discussion

The proposed FFL-IDS provides an effective solution for intrusion detection in environments with decentralized architecture, privacy-preserving techniques, and scalable methodologies. On the other hand, even such frameworks have their limitations. Several challenges are identified, which could further stimulate research on this topic and pave the way for numerous development opportunities. Ultimately, from a computational resource viewpoint, there is a dependency on the edge nodes. IoT equipment in industrial environments oftentimes needs more computational power and memory resources to adequately train CNN models, which significantly deters applicability in resource-constrained or legacy IIoT configurations. Besides, the federated learning approach minimizes the transmission of raw data. However, it inherently generates communication overhead due to periodic exchanges of model updates across edge devices and fog nodes with the global server. This problem worsens in distributed settings, where bandwidth constraints in the network are bound to affect the performance. Advanced adversarial inference attacks are possible threats even after adding privacy-preserving mechanisms, such as the incorporation of Gaussian noise and secure aggregation of FedAvg. Robust privacy against such attacks is a space yet to be explored. Scalability is another big problem, particularly with increases in the scale of participation. The synchronization delay and aggregation bottleneck in large networks could significantly impact the system’s real-time intrusion detection capabilities. The adaptability of the proposed system to novel or zero-day attacks mostly depends on the diversity and quality of training datasets. Regular updates with emerging patterns of threats are necessary to keep the system up-to-date and relevant. There would be an impact on the accurate time detection mainly due to latency in the iteration process of local model training and global aggregation. Accurate time detection can suffer due to high-speed attacks or large data volumes. Non-IID (non-independent and identically distributed) data across participating nodes or imbalanced datasets could have some effects that degrade the system’s performance, limiting generalization towards optimal detection rates. Finally, energy efficiency remains a concern, as continuous model training and communication increase power consumption, posing sustainability challenges for battery-powered IoT devices.

Such constraints will demand more research towards perfecting the application of these resources, scaling their application, and privacy-preserving techniques with much resilience. All these will present gigantic challenges if intrusion detection systems are to be practically positioned within such diverse and emerging IIoT environments. Such implications, like adaptive learning mechanisms, energy-efficient algorithms, and robust data-handling strategies, may make the proposed framework much more viable and impactful.

## 11. Conclusions

In this paper, a Fog-enabled Federated Learning-based Intrusion Detection System (FFL-IDS) is proposed to address the problems of jamming and spoofing attacks in IIoT environments. Federated learning and fog computing is used in the proposed architecture to realize more privacy, scalability, and detection on the fly. This way, the system achieves data privacy protection using high accuracy and efficiency through localized CNN training at the edge devices and model update aggregation at the fog layer. The proposed system was tested on two benchmark datasets: Edge-IIoTset and CIC-IDS2017. The outcomes show the efficiency and robustness of the overall framework with an accuracy of 93.4% for Edge-IIoTset and 95.8% for CIC-IDS2017, respectively, along with high precision, recall, F1 score, and specificity. Such results speak about the portability of the developed framework in various IIoT environments and its ability to combat a significant fraction of threats. Nevertheless, the FFL-IDS framework faces several challenges. Some challenges, for example, were identified in the edge, communication overhead, and scalability bottlenecks in large-scale deployments. Second, the issue of resilience against advanced inference attacks also needs more future research as well as adaptation towards shifting threat landscapes. This work provides the groundwork for developing scalable and privacy-preserving intrusion detection systems for IIoT. Future work can focus on resource utilization optimization, data imbalance at nodes, and system resilience to new attacks. Integrating energy-efficient mechanisms and adaptive learning algorithms can enhance its applicability to highly resource-constrained and dynamic IIoT environments. The FFL-IDS framework provides a significant stepping stone towards IIoT environment’s security and opens new doors for further advancement in this critical area.

## Figures and Tables

**Table 1:** IIoT attack types and potential impacts.

Attack Type	Category	Description	Impact	Citation
DoS	Disruption	Overloads the network with traffic	Downtime	[29]
MitM	Eavesdropping	Intercepts and alters communication	Data theft	[30]
Ransomware	Malware	Encrypts data, demands ransom	Financial loss	[31]
Data Breach	Unauthorized Access	Accesses sensitive information	Data loss	[25]
SQL Injection	Injection Attack	Executes malicious SQL queries	Data manipulation	[32]
Eavesdropping	Passive Attack	Monitors network traffic	Confidentiality breach	[27]
Spoofing	Identity Theft	Impersonates users or devices	Access breach	[8]

**Table 2:** Comparison of intrusion detection techniques for IIoT networks.

Ref.	Technique	Methodology	Results	Limitations
[44]	Signal Consistency Checks	Signal strength and location-based consistency	Effective jamming detection	Environment-dependent, single measurement issue
[45]	Random Forest IDS	Wireless 802.11 IDS	High static accuracy	Limited adaptability to new attacks
[46]	DL Classifiers (Clustering, LSTM, RNN)	Anomaly detection for IIoT	98.8% accuracy with clustering	Computationally heavy
[47]	ML Classifiers	Malware detection in IoT networks	Efficient and lightweight	Limited to malware, single-vector focus
[48]	Hybrid Cu-LSTMGRU + Cu-BLSTM	Hybrid deep learning for IIoT threat mitigation	99.45% accuracy, F1-score 99.47%	Data-intensive, heavy on resources
[50]	A1DE/A2DE Classifiers	DoS attack detection	High precision	Relies on static datasets
[52]	SVM + Feature Selection	Binary classification for IoT NIDS	85.99% accuracy for binary classification	Misses multiclass attack detection
[49]	Federated Learning + Fog CNN	Federated learning with Fog-enabled CNN	Scalable and efficient	Dataset limitations and scalability issues

**Table 3:** Experimental setup.

Simulation Parameters	Value
IDE	Google Colaboratory
Online Storage	Google Drive for dataset
Programming language	Python
Python Version	3.7
Computation	GPU
RAM	12.68 GB
Encoding	One Hot Encoder
Number of GPUs	1
Libraries used	Pandas, NumPy, sklearn, keras
Libraries for FL	Tensorflow, Federated tensorflow
Local Model trained	10
Global Model trained	1

**Table 4:** The total numbers and the different types of records in the Edge-IIoTset dataset.

IoT Traffic	Type of Event	Data Record	Description
Normal	Normal	1,091,198	Standard traffic.
Normal	DDoS-UDP	97,253	UDP network flood.
Normal	DDoS-ICMP	54,351	ICMP traffic flood.
Normal	SQL injection	40,661	SQL query manipulation.
Normal	DDoS-TCP	40,050	Misused TCP connections.
Normal	Vulnerability scanner	40,021	Security weakness scan.
Normal	Password	39,946	Password force attempts.
Normal	DDoS-HTTP	38,835	HTTP service disruption.
Attack	Uploading	29,446	Exploit through uploads.
Attack	Backdoor	19,221	Unauthorized access.
Attack	Port-scanning	15,982	Vulnerability scanning.
Attack	XSS	12,058	Malicious script execution.
Attack	Ransomware	7751	Data hijack for ransom.
Attack	Fingerprinting	6822	System data collection.
Attack	MITM	286	Communication interception.

**Table 5:** Details of the CIC-IDS2017 dataset.

Traffic Type	CIC-IDS2017	Description
BENIGN	2,273,097	Non-malicious internet traffic.
Bot	1966	Botnet traffic emanates from infected machines.
DDoS	128,027	Distributed Denial of Service attacks.
DoS GoldenEye	10,293	GoldenEye was the tool used to perform denial of service.
DoS Hulk	231,073	Denial of Service attack kills the target.
DoS Slowhttptest	5499	Denial of Service exploits the vulnerabilities of web servers.
DoS Slowloris	5796	DoS maintaining connections open.
FTP-PATATOR	7938	FTP servers were attacked by brute force.
Heartbleed	11	Exploiting the vulnerability Heartbleed.
Infiltration	36	Unauthorized access attempts.
PortScan	158,930	Port scan to search for open ports.
SSH-PATATOR	5897	An attack by brute force against SSH servers.
WebAttack BruteForce	1507	Brute-force attack against web applications.
WebAttack SQL Injection	21	Exploiting vulnerability SQL injection.
WebAttack XSS	652	Cross-Site Scripting Attack.

**Table 6:** Performance metrics comparison with the state-of-the-art model.

Dataset	Metric	Base Papers	Proposed Paper	Percentage Difference (Improvement)
Edge-IIoTset	Accuracy	87.37%	97%	10.45%
	Precision	90.65%	93%	2.56%
	Recall	77.73%	96%	20.01%
	F1 Score	86.9%	92.6%	6.35%
CIC-IDS2017	Accuracy	89.23%	95.8%	7.1%
	Precision	92.23%	94.9%	2.67%
	Recall	89.92%	94.5%	4.58%

## Data Availability

The data presented in the study are openly available at https://www.kaggle.com/datasets/mohamedamineferrag/edgeiiotset-cyber-security-dataset-of-iot-iiot (accessed on 1 March 2024) and https://www.kaggle.com/datasets/chethuhn/network-intrusion-dataset (accessed on 1 March 2024).

## References

[1] May M.C., Glatter D., Arnold D., Pfeffer D., Lanza G. (2024). IIoT System Canvas—From architecture patterns towards an IIoT development framework. J. Manuf. Syst..

[2] Singh N., Buyya R., Kim H. (2024). Securing Cloud-Based Internet of Things: Challenges and Mitigations. arXiv.

[3] Hajlaoui R., Moulahi T., Zidi S., El Khediri S., Alaya B., Zeadally S. (2024). Towards smarter cyberthreats detection model for industrial Internet of Things (IIoT) 4.0. J. Ind. Inf. Integr..

[4] Quy V.K., Nguyen D.C., Van Anh D., Quy N.M. (2024). Federated learning for green and sustainable 6G IIoT applications. Internet Things.

[5] Khan W.Z., Rehman M., Zangoti H.M., Afzal M.K., Armi N., Salah K. (2020). Industrial internet of things: Recent advances, enabling technologies and open challenges. Comput. Electr. Eng..

[6] Yazdinejad A. (2024). Secure and Private ML-Based Cybersecurity Framework for Industrial Internet of Things (IIoT). Ph.D. Thesis.

[7] Irum S., Ali A., Khan F.A., Abbas H. (2013). A hybrid security mechanism for intra-WBAN and inter-WBAN communications. Int. J. Distrib. Sens. Netw..

[8] Malik P.K., Sharma R., Singh R., Gehlot A., Satapathy S.C., Alnumay W.S., Pelusi D., Ghosh U., Nayak J. (2021). Industrial Internet of Things and its applications in industry 4.0: State of the art. Comput. Commun..

[9] Moqurrab S.A., Anjum A., Tariq N., Srivastava G. (2022). Instant_anonymity: A lightweight semantic privacy guarantee for 5G-enabled IIoT. IEEE Trans. Ind. Inform..

[10] Ali H., Khan F.A. (2013). Attributed multi-objective comprehensive learning particle swarm optimization for optimal security of networks. Appl. Soft Comput..

[11] Ahmad Z., Shahid Khan A., Wai Shiang C., Abdullah J., Ahmad F. (2021). Network intrusion detection system: A systematic study of machine learning and deep learning approaches. Trans. Emerg. Telecommun. Technol..

[12] Jiang K., Wang W., Wang A., Wu H. (2020). Network intrusion detection combined hybrid sampling with deep hierarchical network. IEEE Access.

[13] Zhang C., Jia D., Wang L., Wang W., Liu F., Yang A. (2022). Comparative research on network intrusion detection methods based on machine learning. Comput. Secur..

[14] Moqurrab S.A., Tariq N., Anjum A., Asheralieva A., Malik S.U., Malik H., Pervaiz H., Gill S.S. (2022). A deep learning-based privacy-preserving model for smart healthcare in Internet of medical things using fog computing. Wirel. Pers. Commun..

[15] Mirza N.A.S., Abbas H., Khan F.A., Al Muhtadi J. Anticipating Advanced Persistent Threat (APT) countermeasures using collaborative security mechanisms. Proceedings of the 2014 International Symposium on Biometrics and Security Technologies (ISBAST).

[16] Antunes R.S., André da Costa C., Küderle A., Yari I.A., Eskofier B. (2022). Federated learning for healthcare: Systematic review and architecture proposal. ACM Trans. Intell. Syst. Technol. (TIST).

[17] Ghimire B., Rawat D.B. (2022). Recent advances on federated learning for cybersecurity and cybersecurity for federated learning for internet of things. IEEE Internet Things J..

[18] Tan Y., Long G., Ma J., Liu L., Zhou T., Jiang J. (2022). Federated learning from pre-trained models: A contrastive learning approach. Adv. Neural Inf. Process. Syst..

[19] Lai F., Dai Y., Singapuram S., Liu J., Zhu X., Madhyastha H., Chowdhury M. Fedscale: Benchmarking model and system performance of federated learning at scale. Proceedings of the International Conference on Machine Learning.

[20] Iqbal U., Tandon A., Gupta S., Yadav A.R., Neware R., Gelana F.W. (2022). A novel secure authentication protocol for IoT and cloud servers. Wirel. Commun. Mob. Comput..

[21] Al Muhtadi J., Alamri R.A., Khan F.A., Saleem K. (2021). Subjective logic-based trust model for fog computing. Comput. Commun..

[22] Hazra A., Donta P.K., Amgoth T., Dustdar S. (2022). Cooperative transmission scheduling and computation offloading with collaboration of fog and cloud for industrial IoT applications. IEEE Internet Things J..

[23] Muzammal S.M., Murugesan R.K., Jhanjhi N.Z., Humayun M., Ibrahim A.O., Abdelmaboud A. (2022). A trust-based model for secure routing against RPL attacks in internet of things. Sensors.

[24] Zhou X., Hu Y., Wu J., Liang W., Ma J., Jin Q. (2022). Distribution bias aware collaborative generative adversarial network for imbalanced deep learning in industrial IoT. IEEE Trans. Ind. Inform..

[25] Ashraf H., Hanif M., Ihsan U., Al-Quayed F., Humayun M., Jhanjhi N. A Secure and Reliable Supply chain management approach integrated with IoT and Blockchain. Proceedings of the 2023 International Conference on Business Analytics for Technology and Security (ICBATS).

[26] Arshad D., Asim M., Tariq N., Baker T., Tawfik H., Al-Jumeily OBE D. (2022). THC-RPL: A lightweight Trust-enabled routing in RPL-based IoT networks against Sybil attack. PLoS ONE.

[27] Khan N., Hamid B., Humayun M., Jhanjhi N., Tahir S. (2024). Information Retrieval from Healthcare Information System. Computational Intelligence in Healthcare Informatics.

[28] Inuwa M.M., Das R. (2024). A comparative analysis of various machine learning methods for anomaly detection in cyber attacks on IoT networks. Internet Things.

[29] Pirayesh H., Zeng H. (2022). Jamming attacks and anti-jamming strategies in wireless networks: A comprehensive survey. IEEE Commun. Surv. Tutorials.

[30] Sen S., Song L. An IIoT-based networked industrial control system architecture to secure industrial applications. Proceedings of the 2021 IEEE Industrial Electronics and Applications Conference (IEACon).

[31] Al-Quayed F., Ahmad Z., Humayun M. (2024). A situation based predictive approach for cybersecurity intrusion detection and prevention using machine learning and deep learning algorithms in wireless sensor networks of industry 4.0. IEEE Access.

[32] Ji Z., Yeoh P.L., Chen G., Zhang J., Zhang Y., He Z., Yin H., Li Y. (2022). Physical-layer-based secure communications for static and low-latency industrial internet of things. IEEE Internet Things J..

[33] Khan I.A., Keshk M., Pi D., Khan N., Hussain Y., Soliman H. (2022). Enhancing IIoT networks protection: A robust security model for attack detection in Internet Industrial Control Systems. Ad Hoc Netw..

[34] Guezzaz A., Azrour M., Benkirane S., Mohy-Eddine M., Attou H., Douiba M. (2022). A lightweight hybrid intrusion detection framework using machine learning for edge-based IIoT security. Int. Arab. J. Inf. Technol..

[35] Altunay H.C., Albayrak Z. (2023). A hybrid CNN+ LSTMbased intrusion detection system for industrial IoT networks. Eng. Sci. Technol. Int. J..

[36] Fu Y., Du Y., Cao Z., Li Q., Xiang W. (2022). A deep learning model for network intrusion detection with imbalanced data. Electronics.

[37] Yang Z., Chen M., Wong K.K., Poor H.V., Cui S. (2022). Federated learning for 6G: Applications, challenges, and opportunities. Engineering.

[38] Schmidl S., Wenig P., Papenbrock T. (2022). Anomaly detection in time series: A comprehensive evaluation. Proc. VLDB Endow..

[39] Zafar A., Aamir M., Mohd Nawi N., Arshad A., Riaz S., Alruban A., Dutta A.K., Almotairi S. (2022). A comparison of pooling methods for convolutional neural networks. Appl. Sci..

[40] Zhang X., Zhang X., Wang W. (2023). Convolutional Neural Network. Intelligent Information Processing with Matlab.

[41] Ali S.E., Tariq N., Khan F.A., Ashraf M., Abdul W., Saleem K. (2023). BFT-IoMT: A blockchain-based trust mechanism to mitigate sybil attack using fuzzy logic in the internet of medical things. Sensors.

[42] Nandanwar H., Katarya R. (2024). Deep learning enabled intrusion detection system for Industrial IOT environment. Expert Syst. Appl..

[43] Hassan M.M., Gumaei A., Huda S., Almogren A. (2020). Increasing the trustworthiness in the industrial IoT networks through a reliable cyberattack detection model. IEEE Trans. Ind. Inform..

[44] Xu W., Trappe W., Zhang Y., Wood T. The feasibility of launching and detecting jamming attacks in wireless networks. Proceedings of the 6th ACM International Symposium on Mobile Ad Hoc Networking and Computing.

[45] Puñal O., Aktaş I., Schnelke C.J., Abidin G., Wehrle K., Gross J. Machine learning-based jamming detection for IEEE 802.11: Design and experimental evaluation. Proceedings of the IEEE International Symposium on a World of Wireless, Mobile and Multimedia Networks 2014.

[46] Bansal A., Mahapatra S. A comparative analysis of machine learning techniques for botnet detection. Proceedings of the 10th International Conference on Security of Information and Networks.

[47] Sharma A., Sahay S.K. (2016). An effective approach for classification of advanced malware with high accuracy. arXiv.

[48] Javeed D., Gao T., Khan M.T., Shoukat D. (2022). A hybrid intelligent framework to combat sophisticated threats in secure industries. Sensors.

[49] Ge M., Syed N.F., Fu X., Baig Z., Robles-Kelly A. (2021). Towards a deep learning-driven intrusion detection approach for Internet of Things. Comput. Netw..

[50] Baig Z.A., Sanguanpong S., Firdous S.N., Vo V.N., Nguyen T.G., So-In C. (2020). Averaged dependence estimators for DoS attack detection in IoT networks. Future Gener. Comput. Syst..

[51] Koroniotis N., Moustafa N., Sitnikova E., Turnbull B. (2019). Towards the development of realistic botnet dataset in the internet of things for network forensic analytics: Bot-iot dataset. Future Gener. Comput. Syst..

[52] Jing D., Chen H.B. SVM based network intrusion detection for the UNSW-NB15 dataset. Proceedings of the 2019 IEEE 13th International Conference on ASIC (ASICON).

[53] Khammassi C., Krichen S. (2020). A NSGA2-LR wrapper approach for feature selection in network intrusion detection. Comput. Netw..

[54] Hassija V., Chamola V., Agrawal A., Goyal A., Luong N.C., Niyato D., Yu F.R., Guizani M. (2021). Fast, reliable, and secure drone communication: A comprehensive survey. IEEE Commun. Surv. Tutor..

[55] Abdulkareem S.A., Foh C.H., Carrez F., Moessner K. (2024). A lightweight SEL for attack detection in IoT/IIoT networks. J. Netw. Comput. Appl..

[56] Truong V.T., Le L.B. (2023). MetaCIDS: Privacy-preserving collaborative intrusion detection for metaverse based on blockchain and online federated learning. IEEE Open J. Comput. Soc..

